# Systemic serological signatures and novel biomarker CST4 link CagA-positive *Helicobacter pylori* to broad systemic alterations

**DOI:** 10.3389/fcimb.2026.1826407

**Published:** 2026-06-26

**Authors:** Chonghe Xu, Shuping Lu, Shulei Zhang, Miaosu Chang, Shuo Zhang, Wei Xu, Mei Zhu

**Affiliations:** 1 School of Basic Medical Sciences, Capital Medical University, Beijing, China; 2 Beijing Anzhen Hospital, Capital Medical University, Beijing, China; 3 Department of Clinical Laboratory, The Fourth Affiliated Hospital (Affiliated Chaohu Hospital) of Anhui Medical University, Chaohu, Anhui, China; 4 Department of Blood Transfusion, The First Affiliated Hospital of Anhui Medical University, Hefei, Anhui, China

**Keywords:** CagA, cystatin 4, *Helicobacter pylori*, nomogram, risk prediction, serological markers, virulence factors

## Abstract

**Objective:**

*Helicobacter pylori (H. pylori)* virulence factors, particularly CagA, determine pathogenic outcomes, yet their systemic serological impact remains poorly characterized. This study aimed to delineate systemic serological signatures associated with CagA-positive *H. pylori* infection, evaluate the novel biomarker CST4 as an exploratory pathophysiological indicator of host response to virulent infection, and develop predictive nomograms integrating host and bacterial factors for gastric diseases.

**Methods:**

This retrospective study enrolled 565 participants who underwent bidirectional endoscopy with histopathological confirmation. *H. pylori* virulence-associated serotypes were identified using immunoblot analysis targeting CagA, VacA, UreA, and UreB proteins. Based on serological results, participants were categorized into three groups: Hp-negative, Hp I (positive for CagA and/or VacA), and Hp II (positive only for UreA and/or UreB). A comprehensive panel of serological parameters was assessed, including gastric function indicators (PG-I, PG-II, PG-I/II ratio, and G-17), inflammatory markers, lipid profiles, coagulation indices, tumor markers, and serum CST4 levels. Multivariate logistic regression analysis was performed to determine independent predictors, which were subsequently used to develop predictive nomograms.

**Results:**

CagA-positive infection, particularly the CagA^+^VacA^+^ subtype, correlated with aggravated mucosal damage (elevated PG-II, reduced PG-I/II ratio, both *P* < 0.001). Serum CST4 levels were significantly elevated in Hp I patients (*P* = 0.032). The CST4-elevated group exhibited increased PG-I, PG-II, CA19-9, CA125, and D-dimer (all *P* < 0.05). CagA-positive infection was characterized by distinct systemic alterations: neutrophilia, elevated D-dimer and creatinine, shortened APTT, and reduced TC, HDL-C, and LDL-C (all *P* < 0.05). Younger age (OR = 0.976) and lower PG-I/II ratio (OR = 0.890) independently predicted Hp I infection. Three nomograms integrating host and bacterial factors demonstrated robust discrimination for gastric polyps (AUC = 0.808), ulcers (AUC = 0.796), and cancer (AUC = 0.709), with favorable calibration and net clinical benefit by decision curve analysis.

**Conclusion:**

CagA-positive *H. pylori* infection induces a distinct systemic serological signature encompassing pro-inflammatory, pro-coagulant, hypolipidemic, and renal alterations, extending pathogenic impact beyond the gastric niche. Serum CST4 emerges as a promising exploratory pathophysiological indicator reflecting host systemic response to virulent infection, supporting its utility in screening and risk triage, while lesion-specific prediction is better achieved by nomograms integrating direct bacterial and host factors. The validated nomograms enable individualized risk stratification, supporting optimized clinical triage in *H. pylori*-infected populations.

## 1 Introduction

*Helicobacter pylori* (*H. pylori*) infection remains one of the most prevalent chronic bacterial infections worldwide, colonizing approximately half of the global population. As the primary etiological agent for chronic gastritis, peptic ulcers, and gastric adenocarcinoma, it is designated a Group I carcinogen by the International Agency for Research on Cancer (IARC) (Hooi et al., 2017). Clinical outcomes of *H. pylori* infection are heterogeneous, ranging from asymptomatic colonization to gastric cancer (GC), driven by the complex interplay of bacterial virulence, host genetics, and environmental exposures (Lin and Koskella, 2015).

Among the virulence factors, the cytotoxin-associated gene A (CagA) protein is a key determinant of pathogenicity, strongly associated with intense gastric inflammation, peptic ulcers, and progression to GC (Beltrán-Anaya et al., 2014). The vacuolating cytotoxin A (VacA) is another major virulence factor contributing to epithelial damage and immune modulation (Holland et al., 2020). Serological detection of CagA and VacA antibodies enables non-invasive risk stratification of patients harboring virulent strains (Castillo-Rojas et al., 2004).

Beyond localized gastric damage, chronic infection with virulent CagA-positive strains exerts profound systemic effects, driving immune dysregulation and elevating the risk of extra-gastric diseases (Chen et al., 2024; Liu et al., 2025). Comprehensively mapping this systemic spillover requires multi-dimensional serological assessment. Locally, gastric function indicators (PG-I, PG-II, PG-I/II ratio, and G-17) reliably evaluate mucosal atrophy and premalignant progression risk (Zagari et al., 2017). Systemically, virulent strains trigger inflammatory and metabolic reprogramming. Routine inflammatory indices (e.g., neutrophil-to-lymphocyte ratio, platelet-to-lymphocyte ratio) and coagulation parameters (e.g., D-dimer, APTT) directly reflect this pro-inflammatory and pro-thrombotic milieu (Consolazio et al., 2004; Al-Shawk et al., 2025; Ye et al., 2025). Concurrently, the pathogen-host metabolic interactions and persistent low-grade inflammation frequently perturb host lipid profiles. Furthermore, traditional tumor markers (e.g., CEA, CA19-9, and CA125) are increasingly recognized not merely as oncological diagnostics, but as sensitive indicators of severe chronic epithelial injury and aberrant mucosal remodeling driven by virulent infection (Kim et al., 2009).

Furthermore, chronic *H. pylori* infection, particularly by virulent CagA-positive strains, frequently triggers aberrant systemic autoimmunity. Persistent antigen exposure induces circulating autoantibodies via molecular mimicry or bystander activation, exacerbating systemic tissue damage. This pathogen-driven immune dysregulation and autoantibody-mediated systemic serological alterations share fundamental pathological mechanisms with other systemic conditions. Analogous autoimmune responses and autoantibody profiles act as critical drivers in IgA nephropathy (Al-Karawi and Kadhim, 2024), Hashimoto’s thyroiditis (Kadhim and Al-Karawi, 2024), severe preeclampsia (Jaber et al., 2024), and chronic inflammatory metabolic syndromes like obesity and type II diabetes (Kadhim and Kadhim, 2024). To accurately capture these widespread physiological disruptions, modern diagnostics increasingly emphasize the integration of multidimensional laboratory parameters. This holistic serological profiling demonstrates significant clinical utility across diverse fields, ranging from monitoring hyperinflammatory responses in acute viral infections (Al-Jindeel et al., 2021) and evaluating renal impairment markers (Asaad et al., 2023), to screening for high-risk maternal infections (Mohammed et al., 2023). By recognizing these shared pathways and employing a comprehensive diagnostic framework, we contextualize virulent *H. pylori* not merely as a localized gastric pathogen, but as a potent disruptor of systemic immune homeostasis.

Within this complex network, accurate non-invasive risk stratification of *H. pylori*-associated sequelae remains challenging. While profiling virulence factors like CagA and VacA provides pathogenic insights, novel host-derived biomarkers reflecting the systemic impact of infection are needed. Cystatin 4 (CST4), a secreted type 2 cysteine protease inhibitor, regulates proteolytic activity by forming reversible tight-binding complexes with cysteine proteases such as cathepsins, thereby maintaining extracellular matrix (ECM) homeostasis and modulating inflammation. In pathological states, dysregulated CST4 expression has been implicated in tumor progression. Mechanistically, CST4 overexpression may suppress cathepsin-mediated degradation of ECM components, paradoxically disrupting normal ECM turnover and creating a permissive microenvironment for tumor cell invasion and dissemination. Additionally, CST4 facilitates epithelial-mesenchymal transition (EMT) by modulating the expression of EMT-related transcription factors, and by altering cell adhesion profiles (Cai et al., 2022; Zhou et al., 2024; Gu et al., 2025). Specifically, its association with *H. pylori* virulence serotypes, and its potential as a systemic indicator reflecting extra-gastric host responses (e.g., coagulation, inflammation, lipid metabolism), remains unexplored.

Furthermore, existing risk stratification tools possess notable limitations. The serological ABC method dichotomizes *H. pylori* status without distinguishing high-risk CagA-positive strains (Zhou et al., 2022; Zhang et al., 2025). Conversely, OLGA/OLGIM histopathological staging is the gold standard but requires invasive biopsy mapping, limiting its utility in primary triage (Sağlam and Civan, 2023). Moreover, these models predominantly target gastric cancer as a single endpoint, largely neglecting benign but burdensome lesions like polyps and ulcers. Crucially, they fail to systematically incorporate systemic biomarkers—such as coagulation and inflammatory indices—that reflect the extra-gastric impact of virulent infection.

To address these gaps, this study aimed to systematically investigate the associations between *H. pylori* virulence serotypes and comprehensive host serological profiles. We sought to characterize the distinct systemic serological signature of CagA-positive infection, specifically evaluating serum CST4 as an exploratory pathophysiological indicator. Ultimately, we aimed to develop and validate nomograms for predicting the risks of gastric polyps, ulcers, and cancer by integrating demographic, virulence serotype, and multi-system serological data. By capturing these systemic signatures, this integrative approach transcends the ‘gastric niche,’ providing robust tools for the individualized, risk-stratified management of *H. pylori*-infected populations.

## 2 Materials and methods

### 2.1 Study design and population

A total of 565 participants with various *H. pylori* infection statuses were retrospectively reviewed at a tertiary hospital in China from January 2021 to December 2024. Although this was a retrospective cohort, the adequacy of the sample size was rigorously verified to ensure statistical robustness. First, for the construction of predictive nomograms via multivariable logistic regression, the sample size strictly adhered to the widely accepted “events per variable” (EPV) criterion. To minimize overfitting, at least 10 events per candidate predictor are required. Our cohort included 112 cases of gastrointestinal cancer, 162 cases of ulcers, and 212 cases of polyps, which abundantly satisfied this requirement. Second, for the comparative analysis of continuous host systemic parameters between the primary groups, a statistical power analysis indicated that the enrolled sample size provided >95% power to detect a clinically meaningful medium effect size (Cohen’s d = 0.3) at a two-sided α level of 0.05. Therefore, the sample size of 565 was deemed highly adequate. Inclusion criteria comprised: (1) complete clinical records; (2) age ≥18 years; (3) receipt of bidirectional endoscopy (gastroscopy followed immediately by colonoscopy); (4) definitive histopathological diagnosis; and (5) confirmed *H. pylori* status. Exclusion criteria were as follows: (1) history of *H. pylori* eradication treatment over the past four weeks; (2) prior history of gastrointestinal surgery, including gastrectomy or colectomy; (3) patients with tumors in other sites; (4) patients who underwent repeated hospitalizations or had a history of endoscopic polyp therapy; (5) presence of severe systemic comorbidities (e.g., end-stage organ failure or advanced extra-gastric malignancies).

### 2.2 Baseline clinical and demographic characteristics

Clinical data were extracted from the Hospital Information System (HIS). Trained general practitioners obtained information regarding age, gender, past medical history, alcohol consumption, smoking status, and family history through standardized interviews. Anthropometric measurements and blood pressure (BP) were recorded by trained nursing staff. Body mass index was calculated as weight in kilograms divided by height in meters squared. BMI was categorized as underweight (< 18.5 kg/m^2^), normal (18.5 ≤ BMI < 25.0 kg/m^2^), overweight (25.0 ≤ BMI < 30.0 kg/m^2^), or obese (≥ 30.0 kg/m^2^) (Zhou et al., 2022).

### 2.3 Blood sample collection and laboratory analysis

Venous blood samples were collected following a 12-hour overnight fast into specific vacuum blood collection tubes to ensure pre-analytical quality. Specifically, ethylenediaminetetraacetic acid dipotassium (EDTA-K2) tubes were used for whole blood cell counts; 3.2% sodium citrate tubes were utilized for coagulation parameters; and serum separator tubes (SST) containing clot activator and gel were employed for the assessment of biochemical indices, gastric function markers, tumor markers, and serum CST4. Biochemical parameters—including fasting glucose (FBG), glycosylated serum protein (GSP), total homocysteine (THCY), alanine aminotransferase (ALT), aspartate aminotransferase (AST), γ-glutamyltransferase (GGT), total bilirubin (TBIL), creatinine (Cr), and HbA1c—were measured using a SIEMENS ADVIA XPT analyzer. Specifically, a comprehensive lipid profile encompassing total cholesterol (TC), triglycerides (TG), high-density lipoprotein cholesterol (HDL-C), and low-density lipoprotein cholesterol (LDL-C) was incorporated to evaluate potential systemic metabolic reprogramming and lipid dyshomeostasis secondary to chronic pathogen-driven inflammation. The gastric function indexes (PG-I, PG-II, PG-I/PG-II and G-17) were detected by Hotgen-C2000 automatic chemiluminescence instrument. Whole blood cells count was performed on a Sysmex XN9000 analyzer. The neutrophil-to-lymphocyte ratio (NLR) and platelet-to-lymphocyte ratio (PLR) were calculated by dividing the absolute neutrophil count and the absolute platelet count by the absolute lymphocyte count, respectively. Coagulation parameters (PT, INR, APTT, FIB, TT, and D-dimer) were assessed using a SYSMEX CS 5100 analyzer. These hematological and coagulation indices were specifically selected to characterize the host’s systemic pro-inflammatory milieu (Sağlam and Civan, 2023) and potential hypercoagulable state (Colucci et al., 2005), both of which are hypothesized to be critical systemic manifestations of virulent *H. pylori* infection beyond the gastric niche (Gravina et al., 2018). The abdominal tumor markers AFP, CEA, CA19-9, CA125, CA15-3, and CA72–4 were detected by electrochemiluminescence immunoassay (Roche Diagnostics, Basel, Switzerland). CST4 levels were detected using a human CST4 ELISA kit (Shanghai Liangrun Biomedical Technology Co., Ltd.). The cut-off value used to indicate positive cancer results was 101.0 U/ml. Accordingly, patients were stratified into a CST4-normal group (≤101 U/mL) and a CST4-elevated group (>101 U/mL) for subsequent comparative analyses.

### 2.4 Subtyping detection of *H. pylori*

Antibodies against specific *H. pylori* antigens, including urease A and B subunits (UreA, UreB), CagA, and VacA, were detected via immunoblotting. In this assay, specific serum IgGs were captured by recombinant antigens immobilized on a nitrocellulose membrane and visualized as distinct bands via an enzyme-mediated colorimetric reaction. These resulting bands identified the target antigens by their specific molecular weights: CagA at 116 kD, VacA at 95 and 91 kD, UreB at 66 kD, and UreA at 30 kD. Based on their distinct pathogenic potentials, the infections were classified into two clinically recognized sub-types: Type I *H. pylori* (Hp I, representing highly virulent strains) infection was defined by CagA and/or VacA positivity, while Type II *H. pylori* (Hp II, representing low-virulence strains) infection was characterized by UreA and/or UreB positivity in the absence of CagA and VacA bands. Subjects negative for all four antibodies were categorized as *H. pylori*-negative. The experiments were conducted strictly following the instructions, using reagents purchased from Shenzhen Boluote Biological Products Co., Ltd.

### 2.5 Upper gastrointestinal endoscopy and histological categories

All endoscopic examinations were performed by experienced endoscopists. Diagnoses-including normal mucosa, polyps, chronic non-atrophic gastritis, atrophic gastritis, ulcers, and malignancies-were established based on endoscopic findings and confirmed histopathologically by two independent pathologists using WHO classification criteria and the updated Sydney System. The discrepancies were resolved through adjudication by a third pathologist. The reference group consisted of individuals with no endoscopic or histopathological evidence of gastrointestinal lesions (including polyps, ulcers, or malignancies), irrespective of their *H. pylori* infection status.

### 2.6 Statistical analysis

Statistical analysis and data visualization were performed using SPSS version 26.0 (IBM Corporation, Armonk, NY, USA), OriginPro 2024 (OriginLab Corporation, Northampton, MA, USA), and R software version 4.5.2 (R Foundation for Statistical Computing, Vienna, Austria). Missing data for serum CST4 (62.5%) and lipid profiles were addressed using Multiple Imputation by Chained Equations (MICE) under the missing at random (MAR) assumption. The imputation model incorporated clinical predictors, disease groupings, and auxiliary variables to generate 40 imputed datasets (60 iterations per chain). Derived indices (e.g., PG-I/PG-II ratio, NLR, and PLR) were calculated passively post-imputation. Final statistical inferences and model estimates were pooled according to Rubin’s rules.

Categorical variables are presented as frequencies and percentages (%). Data normality was assessed using the Kolmogorov–Smirnov test. Continuous variables are presented as mean ± standard deviation (SD) for normally distributed data, or as median and interquartile range (IQR) for non-normally distributed data. Inter-group comparisons utilized one-way ANOVA for normally distributed variables and the Mann–Whitney U or Kruskal–Wallis tests for non-normal distributions. For group comparison figures of non-normally distributed data, box plots were used to display median and IQR, with whiskers extending to 1.5×IQR to reflect data distribution, and outliers plotted as individual points. Categorical variables were compared using the chi-square test or Fisher’s exact test, depending on the expected cell frequencies. Multivariate logistic regression was applied to identify independent predictors, with results reported as adjusted odds ratios (ORs) and 95% confidence intervals (CIs). A nomogram prediction model was then constructed. Internal validation was performed via bootstrapping using the *caret* package, discriminative ability was evaluated by calculating the area under the curve (AUC) with the *pROC* package, and calibration was assessed via calibration plots. A two-tailed p-value of less than 0.05 was considered statistically significant, accounting for multiple comparisons.

## 3 Results

### 3.1 Baseline characteristics

**Table 1** summarizes the baseline demographic and clinical characteristics of the 565 study participants, stratified by disease subtype: reference group (n=79), chronic gastritis/polyp (CG/Polyp, n=212), ulcer (n=162), and gastrointestinal cancer (GC/CRC/EC, n=112). Demographically, sex, age, BMI, smoking and drinking status differed significantly (all *P* < 0.050). Regarding comorbidities, cardiac disease prevalence, age-adjusted Charlson comorbidity index differed significantly (all *P* < 0.001). SBP and DBP also varied significantly (all *P* < 0.010). *H. pylori* infection rates (Hp I/Hp II combined and Hp I alone) were significantly higher in ulcer and cancer groups, as were virulence factors CagA, VacA, UreA, and UreB (all *P* < 0.001).

**Table 1:** Basic characteristics of the study participants.

Patient variable	Overall (n=565)	Reference group (n=79)	CG/polyp (n=212)	Ulcer (n=162)	GC/CRC/EC (n=112)	*χ^2^/F*/*H*	*P*
Male sex, No. (%)	346 (61.24)	45 (56.96)	96 (45.28)	126 (77.78)	79 (70.54)	46.093	0.000
Age years, median (IQR)	65.0 (54.0, 76.0)	76.0 (60.0, 82.0)	60.0 (52.0, 72.8)	58.0 (50.8, 72.0)	71.0 (65.0, 77.0)	64.001	0.000
<40	43 (7.61)	3 (3.80)	18 (8.49)	22 (13.58)	0 (0)	52.882	0.000
40-59	188 (33.27)	16 (20.25)	83 (39.15)	67 (41.36)	22 (19.64)		
≥60	334 (59.12)	60 (75.95)	111 (52.36)	73 (45.06)	90 (80.36)		
BMI (n=425), (mean ± SD)	22.86 ± 3.99	21.36 ± 3.80	22.67 ± 3.77	23.75 ± 3.91	22.88 ± 4.41	3.534	0.015
Underweight (BMI < 18.5), No. (%)	54 (9.56)	8 (10.13)	25 (11.79)	7 (4.32)	14 (12.50)	14.700	0.100
Normal (18.5 ≤ BMI < 25.0), No. (%)	255 (45.13)	20 (25.32)	126 (59.43)	57 (35.19)	52 (46.43)		
Overweight (25.0 ≤ BMI < 30.0), No. (%)	102 (18.05)	7 (8.86)	43 (20.28)	31 (19.14)	21 (18.75)		
Obese (BMI ≥ 30.0), No. (%)	14 (2.48)	0 (0)	4 (1.89)	6 (3.70)	4 (3.57)		
Missing, No. (%)	140 (24.78)	44 (55.70)	14 (6.60)	61 (37.65)	21 (18.75)	/	/
Smoking status, No. (%)							
Former/current	132 (23.36)	11 (13.92)	41 (19.34)	50 (30.86)	30 (26.79)	11.672	0.009
Never	433 (76.64)	68 (86.08)	171 (80.66)	112 (69.14)	82 (73.21)		
Drinking status, No. (%)							
Former/current	108 (19.12)	11 (13.92)	29 (13.68)	42 (25.93)	26 (23.21)	11.506	0.009
Never	457 (80.88)	68 (86.08)	183 (86.32)	120 (74.07)	86 (76.79)		
Hypertension, No. (%)							
Yes	222 (39.29)	34 (43.04)	73 (34.43)	65 (40.12)	50 (44.64)	3.954	0.267
No	343 (60.71)	45 (56.96)	139 (65.57)	97 (59.88)	62 (55.36)		
Cardiac diseases, No. (%)							
Yes	73 (12.92)	20 (25.32)	17 (8.02)	24 (14.81)	12 (10.71)	16.318	0.001
No	492 (87.08)	59 (74.68)	195 (91.98)	138 (85.19)	100 (89.29)		
Diabetes, No. (%)							
Yes	68 (12.04)	14 (17.72)	21 (9.91)	21 (12.96)	12 (10.71)	3.637	0.303
No	497 (87.96)	65 (82.28)	191 (90.09)	141 (87.04)	100 (89.29)		
Age-adjusted Charlson comorbidity index, No. (%)							
0	43 (7.61)	4 (5.06)	35 (16.51)	3 (1.85)	1 (0.89)	88.913	0.000
1	93 (16.46)	6 (7.59)	54 (25.47)	33 (20.37)	0 (0)		
≥2	429 (75.93)	69 (87.34)	123 (58.02)	126 (77.78)	111 (99.11)		
SBP (mmHg), median (IQR)	122.00 (108.00, 136.00)	118.00 (106.00, 139.00)	124.00 (112.00, 138.00)	116.50 (105.00, 132.00)	126.00 (113.25, 136.00)	13.951	0.003
DBP (mmHg), median (IQR)	76.00 (68.00, 84.50)	74.00 (63.00, 81.00)	78.00 (71.00, 87.00)	74.00 (67.00, 81.00)	75.50 (67.00, 82.00)	16.164	0.001
Hp I+Hp II, No. (%)	321 (56.81)	41 (51.90)	97 (45.75)	114 (70.37)	69 (61.61)	24.529	0.000
Hp I, No. (%)	240 (42.48)	28 (35.44)	68 (32.07)	89 (54.94)	55 (49.11)	23.297	0.000
Hp II, No. (%)	81 (14.34)	13 (16.46)	29 (13.68)	25 (15.43)	14 (12.50)	0.829	0.842
CagA (116KD), No. (%)	236 (41.77)	28 (35.44)	66 (31.13)	89 (54.94)	53 (47.32)	24.132	0.000
VacA (91KD), No. (%)	164 (29.03)	16 (20.25)	46 (21.70)	63 (38.89)	39 (34.82)	17.953	0.000
VacA (95KD), No. (%)	164 (29.03)	16 (20.25)	46 (21.70)	63 (38.89)	39 (34.82)	17.953	0.000
UreA (30KD), No. (%)	187 (33.10)	18 (22.78)	48 (22.64)	77 (47.53)	44 (39.29)	31.439	0.000
UreB (66KD), No. (%)	307 (54.34)	39 (49.37)	93 (43.87)	112 (69.14)	63 (56.25)	24.615	0.000
Gastric function indicators (n=535)							
PG-I (ng/mL), median (IQR)	147.90 (87.11,200.00)	148.00 (90.12,200.00)	125.50 (74.95,200.00)	192.70 (122.45,200.00)	136.50 (79.43,200.00)	25.888	0.000
PG-II (ng/mL), median (IQR)	17.40 (9.61, 29.60)	17.42 (8.78, 29.60)	13.21 (8.39, 23.09)	20.73 (12.60, 32.24)	21.25 (11.50, 40.46)	26.794	0.000
PG-I/PG-II, median (IQR)	7.55 (4.88, 11.17)	7.51 (5.46, 14.01)	8.74 (5.96, 12.16)	7.34 (5.37, 10.31)	5.17 (3.67, 8.58)	29.051	0.000
Missing, No. (%)	30 (5.31)	4 (5.06)	11 (5.19)	13 (8.02)	2 (1.79)	/	/
G-17 (pg/mL) (n=554) median (IQR)	57.19 (20.72, 188.23)	88.25 (29.23, 241.88)	47.48 (16.52, 193.90)	53.13 (22.70, 160.10)	61.32 (23.01, 222.18)	5.298	0.151
Missing, No. (%)	11 (1.95)	1 (1.27)	5 (2.36)	3 (1.85)	2 (1.79)	/	/
OB (n=414) positive No. (%)	224 (54.11)	29 (60.42)	56 (33.93)	88 (73.95)	51 (62.20)	48.825	0.000
Missing, No. (%)	151 (26.73)	31 (39.24)	47 (22.17)	43 (26.54)	30 (26.79)	/	/
CST4 (n=212) (U/mL)	47.81 (27.76, 77.81)	41.57 (19.03, 95.90)	41.52 (27.43, 64.47)	44.28 (28.42, 66.56)	58.52 (35.44, 97.05)	5.999	0.112
Missing, No. (%)	353 (62.48)	59 (74.68)	126 (59.43)	120 (74.07)	48 (42.86)	/	/
Abdominal tumor markers (n=521)							
AFP (ng/mL), median (IQR)	2.18 (1.59, 3.09)	2.19 (1.51, 2.88)	2.31 (1.61, 3.18)	2.06 (1.57, 2.90)	2.11 (1.57, 3.41)	4.194	0.241
CEA (ng/mL), median (IQR)	2.02 (1.37, 3.29)	2.04 (1.52, 3.20)	1.89 (1.29, 3.22)	1.82 (1.11, 2.50)	3.15 (1.92, 5.82)	49.617	0.000
CA19-9 (U/mL), median (IQR)	5.87 (3.01, 12.26)	6.78 (3.14, 12.18)	6.07 (3.33, 11.61)	4.48 (2.68, 9.82)	8.18 (3.21, 43.50)	14.755	0.002
CA125 (U/mL), median (IQR)	12.00 (8.40, 19.45)	17.40 (10.10, 30.80)	11.30 (7.95, 17.10)	9.60 (6.70, 16.80)	16.20 (10.30, 25.20)	49.552	0.000
CA15-3 (U/mL), median (IQR)	8.30 (6.10, 11.85)	7.80 (5.50, 12.10)	8.90 (6.95, 13.20)	6.90 (4.80, 9.73)	8.70 (6.70, 12.50)	31.503	0.000
CA72-4 (U/mL), median (IQR)	1.14 (0.41, 2.21)	0.84 (0.20, 1.95)	1.14 (0.36, 2.40)	1.00 (0.41, 1.83)	1.50 (0.71, 3.37)	17.732	0.000
Missing, No. (%)	44 (7.79)	4 (5.06)	19 (8.96)	12 (7.41)	9 (8.04)	/	/
Blood routine tests (n=564)							
PLT ×10^9/L, median (IQR)	177.00 (136.00, 235.00)	158.00 (88.00, 219.00)	182.00 (143.00, 232.00)	177.00 (136.75, 237.25)	196.00 (145.00, 266.00)	15.081	0.002
NEUT ×10^9/L, median (IQR)	4.17 (2.97, 5.84)	4.79 (3.06, 6.75)	3.50 (2.58, 4.66)	5.18 (3.79, 7.62)	3.94 (2.81, 5.30)	63.257	0.000
LYC, median (IQR)	1.31 (0.97, 1.75)	1.01 (0.74, 1.46)	1.34 (1.05, 1.77)	1.50 (1.07, 1.91)	1.19 (0.91, 1.69)	31.859	0.000
PLR, median (IQR))	133.88 (98.28, 198.45)	140.40 (90.36, 229.93)	130.89 (99.27, 180.00)	119.68 (84.35, 181.09)	158.66 (108.10, 236.34)	15.820	0.001
NLR, median (IQR)	3.10 (2.11, 4.91)	4.34 (2.78, 7.26)	2.50 (1.77, 3.54)	3.46 (2.22, 5.50)	3.23 (2.20, 4.93)	43.568	0.000
Missing, No. (%)	1 (0.18)	0 (0.00)	1 (0.47)	0 (0.00)	0 (0.00)	/	/
FBG (n=546) (mmol/L) median (IQR)	5.30 (4.80, 6.50)	5.50 (4.90, 7.35)	5.10 (4.70, 5.70)	5.90 (4.90, 7.00)	5.20 (4.70, 6.20)	25.601	0.000
Missing, No. (%)	19 (3.36)	3 (3.80)	11 (5.19)	3 (1.85)	2 (1.79)	/	/
Serum lipid parameters (n=284)							
GSP (μmol/L), median (IQR)	178.00 (148.00, 204.00)	171.00 (145.50, 197.00)	191.00 (171.00, 217.00)	159.00 (139.00, 192.00)	166.00 (140.50, 204.00)	27.522	0.000
TC (mmol/L), median (IQR)	3.76 (3.11, 4.62)	3.65 (2.88, 4.45)	4.42 (3.56, 5.19)	3.31 (2.89, 3.84)	3.73 (3.10, 4.48)	45.859	0.000
TG (mmol/L), median (IQR)	1.36 (0.94, 1.88)	1.22 (0.83, 1.56)	1.40 (0.91, 1.89)	1.56 (1.07, 2.30)	1.14 (0.91, 1.62)	14.916	0.002
HDL-C (mmol/L), median (IQR)	0.84 (0.62, 1.12)	0.84 (0.56, 1.05)	0.99 (0.75, 1.29)	0.68 (0.53, 0.88)	0.80 (0.60, 1.10)	39.415	0.000
LDL-C (mmol/L), median (IQR)	2.11 (1.50, 2.72)	1.91 (1.44, 2.51)	2.42 (1.83, 3.14)	1.77 (1.32, 2.15)	2.18 (1.60, 2.68)	32.883	0.000
THCY (μmol/L), median (IQR)	13.00 (10.43, 16.75)	14.50 (11.65, 19.40)	13.00 (9.90, 16.50)	12.60 (10.20, 16.30)	13.40 (10.80, 16.05)	4.794	0.188
Missing, No. (%)	281 (49.73)	38 (48.10)	97 (45.75)	83 (51.23)	63 (56.25)	/	/
Liver and kidney function indicators (n=558)							
Cr (µmol/L), median (IQR)	68.00 (56.00, 84.00)	71.50 (58.75, 96.25)	62.00 (53.00, 78.00)	75.00 (60.50, 90.50)	69.00 (56.00, 83.00)	30.957	0.000
ALT (U/L), median (IQR)	18.00 (13.00, 28.00)	20.00 (14.75, 30.25)	19.00 (13.00, 30.00)	19.00 (14.00, 25.75)	15.00 (11.00, 23.00)	10.419	0.015
AST (U/L), median (IQR)	22.00 (18.00, 28.25)	26.00 (21.00, 33.25)	22.00 (18.00, 28.00)	21.50 (17.00, 28.00)	21.00 (17.00, 28.00)	15.251	0.002
GGT (U/L), median (IQR)	16.00 (11.00, 27.00)	18.50 (11.00, 38.50)	16.00 (11.00, 30.00)	16.00 (11.00, 24.00)	16.00 (12.00, 25.00)	2.040	0.564
TBIL (µmol/L), median (IQR)	13.00 (9.00, 18.00)	15.65 (9.78, 24.18)	15.00 (11.00, 19.00)	11.00 (7.33, 14.20)	13.00 (10.00, 19.00)	42.207	0.000
Missing, No. (%)	7 (1.24)	1 (1.27)	3 (1.42)	2 (1.23)	1 (0.89)	/	/
Coagulation function parameters (n=520)							
PT (s), median (IQR)	10.80 (10.20, 11.50)	11.40 (10.60, 12.50)	10.70 (10.00, 11.10)	10.90 (10.40, 11.70)	10.80 (10.20, 11.50)	28.488	0.000
PT%, median (IQR)	93.40 (86.20, 101.70)	86.70 (75.85, 96.23)	95.20 (90.90, 104.80)	92.60 (84.75, 99.75)	93.90 (87.00, 101.25)	30.829	0.000
PT-INR, median (IQR)	0.92 (0.87, 0.99)	0.98 (0.91, 1.07)	0.91 (0.86, 0.95)	0.94 (0.89, 0.99)	0.92 (0.87, 0.99)	33.921	0.000
APTT (s), median (IQR)	26.50 (24.20, 28.88)	26.50 (24.20, 28.73)	27.05 (25.60, 29.60)	25.10 (22.95, 28.00)	26.20 (24.30, 29.00)	26.588	0.000
FIB (g/L), median (IQR)	2.96 (2.37, 3.70)	3.16 (2.15, 3.93)	2.90 (2.44, 3.56)	2.61 (2.09, 3.32)	3.45 (2.83, 4.11)	34.580	0.000
TT (s), median (IQR)	17.85 (16.93, 18.70)	18.00 (17.05, 19.10)	18.10 (17.23, 18.80)	17.80 (16.90, 18.75)	17.50 (16.60, 18.10)	21.614	0.000
D-D (μg/mL), median (IQR)	0.38 (0.18, 0.90)	0.58 (0.38, 1.28)	0.29 (0.16, 0.65)	0.28 (0.15, 0.85)	0.58 (0.30, 1.37)	45.323	0.000
Missing, No. (%)	45 (7.96)	9 (11.39)	20 (9.43)	9 (5.56)	7 (6.25)	/	/

Gastric function markers PG-I, PG-II, and PG-I/PG-II ratio all differed significantly (all *P* < 0.001). Fecal OB positivity was highest in ulcer and cancer groups (*P* < 0.001). Tumor markers CEA, CA19-9, CA125, CA15-3, and CA72–4 showed significant differences (all *P* < 0.001), with cancer patients generally exhibiting highest levels. Blood parameters including PLT, NEUT, LYC, PLR, NLR, and FBG (all *P* < 0.010) differed significantly. Serum lipids (GSP, TC, TG, HDL-C, LDL-C) all differed significantly (all *P* < 0.010). Liver and kidney function indicators Cr, ALT, AST, and TBIL differed significantly (all *P* < 0.050). All coagulation parameters (PT, PT%, PT-INR, APTT, FIB, TT, D-D) showed significant differences (all *P* < 0.010). These findings confirmed substantial heterogeneity among subgroups, supporting stratified analyses in subsequent evaluations.

Stratification by *H. pylori* serostatus revealed significant inter-group variations in demographics, gastric function, and specific biomarkers (**Table 2**). Compared to the Hp-negative and Hp II cohorts, the Hp I group exhibited a higher prevalence of males (*P* = 0.010) and smokers (*P* = 0.001). Notably, Hp I infection was associated with aggravated mucosal impairment, as evidenced by significantly elevated PG-II and reduced PGI/PGII ratios (*P* < 0.001). CST4 levels differed significantly across groups (*P* = 0.032), highest in Hp I-positive population. CA-125 levels varied significantly among the three groups (*P* = 0.002).

**Table 2:** Baseline characteristics of participants with different *H. pylori* infection status.

Patient variable	*H. pylori*–negative (n=244)	Hp I (n=240)	HpII (n=81)	*χ^2^*/*t*/*H*	*P*
Male sex, No. (%)	132 (54.10)	160 (66.67)	54 (66.67)	9.225	0.010
*χ^2^*	9.225	5.178	1.174	/	/
*P*	0.002	0.023	0.279	/	/
Age, median (IQR)	67.0 (55.0, 77.0)	64.0 (54.0, 73.0)	66.0 (54.0, 77.0)	5.876	0.053
<40	17 (6.97)	20 (8.33)	6 (7.41)	2.971	0.563
40-59	73 (29.92)	85 (35.42)	30 (37.04)		
≥60	154 (63.11)	135 (56.25)	45 (55.56)		
*χ^2^*	2.850	1.441	0.610	/	/
*P*	0.241	0.486	0.737	/	/
BMI (n=425), (mean ± SD)	22.58 ± 3.77	23.20 ± 4.17	22.75 ± 4.10	1.126	0.325
Underweight (BMI < 18.5), No. (%)	26 (13.68)	19 (10.56)	9 (16.36)	4.031	0.673
Normal (18.5 ≤ BMI < 25.0), No. (%)	118 (62.11)	107 (59.44)	30 (54.55)		
Overweight (25.0 ≤ BMI < 30.0), No. (%)	41 (21.58)	46 (25.56)	15 (27.27)		
Obese (BMI ≥ 30.0), No. (%)	5 (2.63)	8 (4.44)	1 (1.82)		
*χ^2^*	1.810	2.721	1.678	/	/
*P*	0.613	0.437	0.642	/	/
Missing, No. (%)	54 (22.13)	60 (25.00)	26 (32.10)	/	/
Smoking status, No. (%)					
Former/current	44 (18.03)	74 (30.83)	14 (17.28)	13.024	0.001
Never	200 (81.97)	166 (69.17)	67 (82.72)		
*χ^2^*	6.814	13.005	1.952	/	/
*P*	0.009	0.000	0.162	/	/
Drinking status, No. (%)					
Former/current	39 (15.98)	55 (22.92)	14 (17.28)	3.967	0.138
Never	205 (84.02)	185 (77.08)	67 (82.72)		
*χ^2^*	2.724	3.900	0.205	/	/
*P*	0.099	0.048	0.651	/	/
Hypertension, No. (%)					
Yes	101 (41.39)	90 (37.50)	31 (38.27)	0.810	0.667
No	143 (58.61)	150 (62.50)	50 (61.73)		
*χ^2^*	0.795	0.562	0.041	/	/
*P*	0.373	0.454	0.839	/	/
Cardiac disease, No. (%)					
Yes	30 (12.30)	30 (12.50)	13 (16.05)	0.827	0.661
No	214 (87.70)	210 (87.50)	68 (83.95)		
*χ^2^*	0.149	0.066	0.823	/	/
*P*	0.699	0.798	0.364	/	/
Diabetes, No. (%)					
Yes	24 (9.84)	31 (12.92)	13 (16.05)	2.524	0.283
No	220 (90.16)	209 (87.08)	68 (83.95)		
*χ^2^*	1.962	0.306	1.439	/	/
*P*	0.161	0.580	0.230	/	/
Age-adjusted Charlson comorbidity index, No. (%)					
0	26 (10.66)	11 (4.58)	6 (7.41)	8.172	0.085
1	33 (13.52)	45 (18.75)	15 (18.52)		
≥2	185 (75.82)	184 (76.67)	60 (74.07)		
*χ^2^*	7.482	6.383	0.291	/	/
*P*	0.024	0.041	0.864	/	/
SBP (mmHg), median (IQR)	124.00 (109.25,138.00)	120.50 (107.00,134.00)	121.00 (108.50,133.00)	2.661	0.264
DBP (mmHg), median (IQR)	77.00 (68.25,83.75)	75.00 (68.00,82.75)	76.00 (67.00,85.50)	1.003	0.606
Gastric function indicators (n=535)					
PG-I (ng/mL), median (IQR)	140.85 (81.56,200.00)	154.95 (90.93,200.00)	148.70 (88.70,200.00)	0.733	0.693
PG-II (ng/mL), median (IQR)	12.77 (7.98,24.70)	21.67 (13.36,32.79)	16.60 (8.47,29.60)	31.206	0.000
PG-I/PG-II, median (IQR)	8.87 (6.12,12.70)	6.43 (4.22,9.05)	7.84 (4.67,11.53)	32.785	0.000
Missing, No. (%)	14 (5.74)	14 (5.83)	2 (2.47)	/	/
G-17 (pg/mL) (n=554) median (IQR)	51.80 (15.34,166.05)	58.61 (24.08,186.25)	57.62 (23.06,250.48)	2.988	0.225
Missing, No. (%)	6 (2.46)	4 (1.67)	1 (1.23)	/	/
OB (n=414) positive No. (%)	84 (45.7)	109 (61.2)	31 (59.6)	9.575	0.008
Missing, No. (%)	60 (24.59)	62 (25.83)	29 (35.80)	/	/
CST4 (n=212) (U/mL)	41.64 (22.31,70.93)	51.09 (32.16,91.71)	45.32 (30.59,80.15)	6.892	0.032
Missing, No. (%)	154 (63.11)	139 (57.92)	60 (74.07)	/	/
Abdominal tumor markers (n=521)					
AFP (ng/mL), median (IQR)	2.34 (1.69,3.23)	2.11 (1.57,3.00)	2.03 (1.53,2.99)	2.975	0.226
CEA (ng/mL), median (IQR)	2.16 (1.37,3.29)	2.00 (1.44,3.42)	1.96 (1.18,3.11)	1.343	0.511
CA19-9 (U/mL), median (IQR)	5.98 (3.21,12.96)	5.58 (2.92,11.13)	6.39 (2.49,12.95)	0.760	0.684
CA125 (U/mL), median (IQR)	13.60 (8.40,22.30)	10.80 (7.80,16.30)	13.45 (8.98,20.55)	12.114	0.002
CA15-3 (U/mL), median (IQR)	8.50 (6.30,12.10)	8.00 (6.00,12.10)	7.90 (5.53,10.60)	3.332	0.189
CA72-4 (U/mL), median (IQR)	1.15 (0.32,2.02)	1.15 (0.49,2.28)	0.82 (0.32,2.52)	1.039	0.595
Missing, No. (%)	18 (7.38)	21 (8.75)	5 (6.17)	/	/
Blood routine tests (n=564)					
PLT ×10^9/L, median (IQR)	188.50 (143.00,240.75)	170.00 (129.00,232.00)	186.00 (140.50,246.50)	4.613	0.100
NEUT ×10^9/L, median (IQR)	3.96 (2.75,5.62)	4.33 (3.04,6.16)	4.17 (3.42,6.10)	3.964	0.138
LYC, median (IQR)	1.33 (0.99,1.76)	1.30 (0.96,1.75)	1.33 (0.95,1.80)	0.073	0.964
PLR, median (IQR))	135.38 (99.34,195.46)	129.73 (91.94,194.74)	139.68 (109.38,215.06)	1.576	0.455
NLR, median (IQR)	3.10 (1.85,4.85)	3.01 (2.19,4.92)	3.18 (2.33,5.32)	2.424	0.298
Missing, No. (%)	0 (0.00)	1 (0.42)	0 (0.00)	/	/
FBG (n=546) (mmol/L) median (IQR)	5.30 (4.70,6.20)	5.40 (4.80,6.70)	5.40 (4.90,6.70)	3.128	0.209
Missing, No. (%)	11 (4.51)	6 (2.50)	2 (2.47)	/	/
Serum lipid parameters (n=284)					
GSP (μmol/L), median (IQR)	185.00 (156.00,205.75)	170.00 (146.50,208.50)	173.00 (136.00,191.00)	4.950	0.084
TC (mmol/L), median (IQR)	3.90 (3.21,4.63)	3.64 (3.06,4.51)	3.61 (2.90,4.80)	3.128	0.209
TG (mmol/L), median (IQR)	1.39 (0.94,1.84)	1.35 (0.93,2.12)	1.35 (0.94,1.65)	0.401	0.818
HDL-C (mmol/L), median (IQR)	0.90 (0.67,1.20)	0.82 (0.59,1.07)	0.78 (0.64,0.92)	5.301	0.071
LDL-C (mmol/L), median (IQR)	2.20 (1.71,2.75)	1.92 (1.39,2.59)	2.15 (1.50,3.15)	4.537	0.103
THCY (μmol/L), median (IQR)	14.20 (10.20,17.28)	12.90 (10.40,15.70)	12.50 (11.10,15.30)	2.777	0.249
Missing, No. (%)	120 (49.18)	123 (51.25)	38 (46.91)	/	/
Liver and kidney function indicators (n=558)					
Cr (µmol/L), median (IQR)	66.00 (54.00,84.00)	71.00 (57.00,84.00)	69.00 (60.00,88.50)	3.129	0.209
ALT (U/L), median (IQR)	18.00 (13.00,26.00)	18.50 (13.00,30.00)	18.00 (13.50,25.00)	0.609	0.737
AST (U/L), median (IQR)	23.00 (18.00,29.00)	22.00 (18.00,29.00)	21.00 (18.00,26.00)	1.098	0.577
GGT (U/L), median (IQR)	17.00 (11.00,27.00)	16.00 (11.00,25.00)	17.00 (12.00,27.50)	1.538	0.464
TBIL (µmol/L), median (IQR)	13.20 (9.85,18.00)	13.00 (9.00,18.60)	13.00 (9.00,18.50)	0.037	0.982
Missing, No. (%)	3 (1.23)	4 (1.67)	0 (0.00)	/	/
Coagulation function parameters (n=520)					
PT (s), median (IQR)	10.80 (10.20,11.53)	10.80 (10.20,11.50)	10.90 (10.50,11.80)	2.284	0.319
PT%, median (IQR)	93.40 (85.10,100.80)	94.70 (88.50,103.20)	92.10 (84.40,97.90)	5.878	0.053
PT-INR, median (IQR)	0.93 (0.88,1.00)	0.92 (0.87,0.97)	0.94 (0.90,1.00)	5.171	0.075
APTT (s), median (IQR)	26.80 (24.80,29.20)	26.20 (23.90,28.40)	26.50 (24.10,28.80)	4.386	0.112
FIB (g/L), median (IQR)	3.00 (2.43,3.71)	2.90 (2.37,3.68)	2.95 (2.11,3.76)	0.803	0.669
TT (s), median (IQR)	17.90 (16.90,18.63)	17.80 (16.90,18.80)	17.90 (17.10,18.80)	1.222	0.543
D-D (μg/mL), median (IQR)	0.41 (0.21,1.03)	0.31 (0.17,0.77)	0.40 (0.19,1.02)	6.347	0.042
Missing, No. (%)	18 (7.38)	17 (7.08)	10 (12.35)	/	/

### 3.2 Distribution of *H. pylori* antibody profiles

Antibody profiles were classified into two primary groups based on virulence factor expression: Hp I (n=240; CagA^+^ and/or VacA^+^) and Hp II (n=81; CagA^-^VacA^-^). The Hp I group was further stratified into a double-positive subgroup (CagA^+^VacA^+^, n=160) and a single-positive subgroup (CagA+/VacA+, n=80) as detailed in **Supplementary Table 1**. Within the Hp I group, the predominant profile was CagA^+^VacA^+^UreA^+^UreB^+^ (58.75%, 141/240). In the Hp II group, the CagA^-^VacA^-^UreA^-^UreB^+^ profile predominated (76.54%, 62/81), followed by CagA^-^VacA^-^UreA^+^UreB^+^ subtype (23.46%, 19/81).

### 3.3 Comparison of baseline characteristics by gender

Baseline characteristics stratified by gender (**Table 3**) revealed distinct demographic and clinical patterns. Significant differences were observed in several variables. Males exhibited significantly higher rates of smoking (38.15%) and alcohol consumption (31.21%) compared to females (*P* < 0.001). Seropositivity for *H. pylori* (total and Hp I subtype) and associated virulence antibodies (CagA, UreA, UreB) were significantly more prevalent in males (*P* < 0.05). In terms of laboratory markers, males showed significantly higher PG-I and PG-II, fecal OB positivity, absolute neutrophil counts, NLR, and creatinine, alongside altered lipid profiles and coagulation parameters (*P* < 0.05). Conversely, parameters such as BMI, VacA status, PG-I/PG-II ratio, G-17, and most tumor markers remained comparable between genders.

**Table 3:** Comparison of patient variables between male and female groups.

Patient variable	Male (n=346)	Female (n=219)	*χ^2^*/*t*/*Z*	*P*
Age years, median (IQR)	66.0 (54.0,76.0)	65.0 (54.0,76.0)	-0.225	0.822
<40	33 (9.54)	10 (4.57)	4.975	0.083
40-59	110 (31.79)	78 (35.62)		
≥60	203 (58.67)	131 (59.82)		
BMI (n=425), (mean ± SD)	22.89 ± 4.01	22.83 ± 3.96	0.144	0.885
Underweight (BMI < 18.5), No. (%)	30 (12.35)	24 (13.19)	0.358	0.949
Normal (18.5 ≤ BMI < 25.0), No. (%)	145 (59.67)	110 (60.44)		
Overweight (25.0 ≤ BMI < 30.0), No. (%)	59 (24.28)	43 (23.63)		
Obese (BMI ≥ 30.0), No. (%)	9 (3.70)	5 (2.75)		
Missing, No. (%)	103 (29.77)	37 (16.89)	/	/
Smoking status, No. (%)				
Former/current	132 (38.15)	0 (0.00)	109.019	0.000
Never	214 (61.85)	219 (100.00)		
Drinking status, No. (%)				
Former/current	108 (31.21)	0 (0.00)	84.513	0.000
Never	238 (68.79)	219 (100.00)		
Hypertension, No. (%)				
Yes	139 (40.17)	83 (37.90)	0.291	0.590
No	207 (59.83)	136 (62.10)		
Cardiac diseases, No. (%)				
Yes	52 (15.03)	21 (9.59)	3.527	0.060
No	294 (84.97)	198 (90.41)		
Diabetes, No. (%)				
Yes	41 (11.85)	27 (12.33)	0.029	0.865
No	305 (88.15)	192 (87.67)		
Age-adjusted Charlson comorbidity index, No. (%)				
0	18 (5.20)	25 (11.42)	9.647	0.008
1	52 (15.03)	41 (18.72)		
≥2	276 (79.77)	153 (69.86)		
SBP (mmHg), median (IQR)	120.00 (108.00,133.00)	125.00 (109.00,140.00)	-2.059	0.039
DBP (mmHg), median (IQR)	76.00 (68.00,83.00)	77.00 (69.00,85.00)	-1.236	0.217
Hp I+Hp II, No. (%)	214 (61.85)	107 (48.86)	9.225	0.002
Hp I, No. (%)	160 (46.24)	80 (36.53)	5.178	0.023
Hp II, No. (%)	54 (15.61)	27 (12.33)	1.174	0.279
CagA (116KD), No. (%)	158 (45.66)	78 (35.62)	5.567	0.018
VacA (91KD), No. (%)	108 (31.21)	56 (25.57)	2.073	0.150
VacA (95KD), No. (%)	108 (31.21)	56 (25.57)	2.073	0.150
UreA (30KD), No. (%)	128 (36.99)	59 (26.94)	6.122	0.013
UreB (66KD), No. (%)	202 (58.38)	105 (47.95)	5.887	0.015
Gastric function indicators (n=535)				
PG-I (ng/mL), median (IQR)	160.10 (98.55,200.00)	127.80 (77.41,200.00)	-2.954	0.003
PG-II (ng/mL), median (IQR)	19.48 (10.98,31.24)	15.37 (8.43,26.81)	-2.786	0.005
PG-I/PG-II, median (IQR)	7.33 (4.65,10.96)	8.19 (5.23,11.89)	-1.590	0.112
Missing, No. (%)	18 (5.20)	12 (5.48)	/	/
G-17 (pg/mL) (n=554), median (IQR)	53.42 (20.75,150.38)	65.13 (20.68,226.53)	-1.558	0.119
Missing, No. (%)	6 (1.73)	5 (2.28)	/	/
OB (n=414), positive No. (%)	156 (62.65)	68 (41.21)	18.368	0.000
Missing, No. (%)	97 (28.03)	54 (24.66)	/	/
CST4 (n=212) (U/mL)	49.68 (29.44,85.31)	43.16 (27.08,71.23)	-1.300	0.194
Missing, No. (%)	216 (62.43)	137 (62.56)	/	/
Abdominal tumor markers (n=521)				
AFP (ng/mL), median (IQR)	2.28 (1.63,3.26)	2.01 (1.54,2.82)	-2.139	0.032
CEA (ng/mL), median (IQR)	2.12 (1.42,3.40)	1.90 (1.35,2.99)	-1.371	0.170
CA19-9 (U/mL), median (IQR)	5.62 (3.07,12.16)	6.28 (2.93,13.14)	-0.683	0.495
CA125 (U/mL), median (IQR)	11.50 (7.90,19.40)	12.85 (8.88,19.90)	-1.113	0.266
CA15-3 (U/mL), median (IQR)	7.90 (5.70,11.50)	8.50 (6.90,12.50)	-2.762	0.006
CA72-4 (U/mL), median (IQR)	1.23 (0.44,2.22)	1.07 (0.34,2.12)	-1.314	0.189
Missing, No. (%)	27 (7.80)	17 (7.76)	/	/
Blood routine tests (n=564)				
PLT ×10^9/L, median (IQR)	175.50 (131.75,233.00)	183.50 (142.75,243.25)	-0.814	0.416
NEUT ×10^9/L, median (IQR)	4.60 (3.22,6.73)	3.60 (2.53,4.71)	-5.732	0.000
LYC, median (IQR)	1.31 (0.93,1.80)	1.33 (1.06,1.69)	-0.691	0.490
PLR, median (IQR))	133.40 (96.13,207.21)	134.93 (99.21,189.63)	-0.031	0.975
NLR, median (IQR)	3.49 (2.25,5.64)	2.67 (1.75,3.76)	-5.116	0.000
Missing, No. (%)	0 (0.00)	1 (0.46)	/	/
FBG (n=546) (mmol/L) median (IQR)	5.40 (4.80,6.60)	5.30 (4.80,6.28)	-0.442	0.658
Missing, No. (%)	8 (2.31)	11 (5.02)	/	/
Serum lipid parameters (n=284)				
GSP (μmol/L), median (IQR)	162.00 (142.00,194.00)	195.00 (171.00,216.00)	-5.342	0.000
TC (mmol/L), median (IQR)	3.47 (2.94,4.22)	4.30 (3.56,5.04)	-5.821	0.000
TG (mmol/L), median (IQR)	1.35 (0.97,2.06)	1.36 (0.91,1.73)	-1.311	0.190
HDL-C (mmol/L), median (IQR)	0.74 (0.56,0.99)	1.02 (0.75,1.28)	-6.041	0.000
LDL-C (mmol/L), median (IQR)	1.92 (1.34,2.48)	2.28 (1.93,3.03)	-4.681	0.000
THCY (μmol/L), median (IQR)	13.80 (11.35,17.50)	11.70 (9.40,15.10)	-4.087	0.000
Missing, No. (%)	173 (50.00)	108 (49.32)	/	/
Liver and kidney function indicators (n=558)				
Cr (µmol/L), median (IQR)	75.00 (65.00,89.00)	57.00 (48.00,68.00)	-10.152	0.000
ALT (U/L), median (IQR)	20.00 (14.00,29.00)	16.00 (12.00,23.00)	-3.361	0.001
AST (U/L), median (IQR)	22.00 (18.00,29.00)	22.00 (18.00,27.00)	-0.612	0.540
GGT (U/L), median (IQR)	18.00 (13.00,30.00)	13.00 (10.00,22.00)	-5.165	0.000
TBIL (µmol/L), median (IQR)	13.00 (8.60,19.00)	13.20 (10.00,18.00)	-0.850	0.395
Missing, No. (%)	3 (0.87)	4 (1.83)	/	/
Coagulation function parameters (n=520)				
PT (s), median (IQR)	10.90 (10.30,11.70)	10.60 (10.10,11.30)	-3.498	0.000
PT%, median (IQR)	92.60 (84.40,100.20)	95.20 (89.00,103.20)	-3.304	0.001
PT-INR, median (IQR)	0.94 (0.89,1.00)	0.91 (0.86,0.97)	-3.762	0.000
APTT (s), median (IQR)	26.40 (24.00,29.10)	26.50 (24.40,28.50)	-0.097	0.923
FIB (g/L), median (IQR)	2.90 (2.29,3.70)	3.01 (2.47,3.69)	-1.080	0.280
TT (s), median (IQR)	17.80 (16.90,18.70)	17.90 (17.00,18.75)	-0.797	0.426
D-D (μg/mL), median (IQR)	0.37 (0.18,0.86)	0.39 (0.20,0.98)	-1.397	0.162
Missing, No. (%)	27 (7.80)	18 (8.22)	/	/

### 3.4 Association between *H. pylori* antibody profiles and clinical diagnosis

The distribution of *H. pylori* antibody profiles across clinical diagnostic groups is summarized in **Supplementary Table 2**. Within the Hp I cohort (n=240), the double-positive CagA^+^VacA^+^ subtype was significantly associated with clinical pathologies (*P* < 0.001), particularly the ulcer group (38.89%). The predominant comprehensive profile, CagA^+^VacA^+^UreA^+^UreB^+^ (24.96%), was similarly concentrated in the ulcer group (35.19%). Conversely, neither the CagA^+^/VacA^+^ status nor the Hp II profile showed significant inter-group differences (*P* = 0.515 and *P* = 0.842, respectively).

### 3.5 Association between patient variables and seropositivity of *H. pylori* virulence factors

Seropositivity for *H. pylori* virulence factors significantly correlated with various host characteristics (**Supplementary Table 3**). Specifically, age and smoking status were consistently associated with higher virulence factor seroprevalence (*P* < 0.05). Virulence seropositivity was robustly linked to elevated PG-II and a diminished PG-I/PG-II ratio (all *P* < 0.001), indicating aggravated mucosal inflammation. Positive fecal occult blood was associated with CagA and UreB. Several coagulation parameters (PT%, PT-INR, APTT, TT, DD) also showed significant associations with specific virulence factors, underscoring a complex host-pathogen interaction.

### 3.6 Comparison of serum gastric biomarker levels between CST4 normal and elevated groups

**Figure 1:**
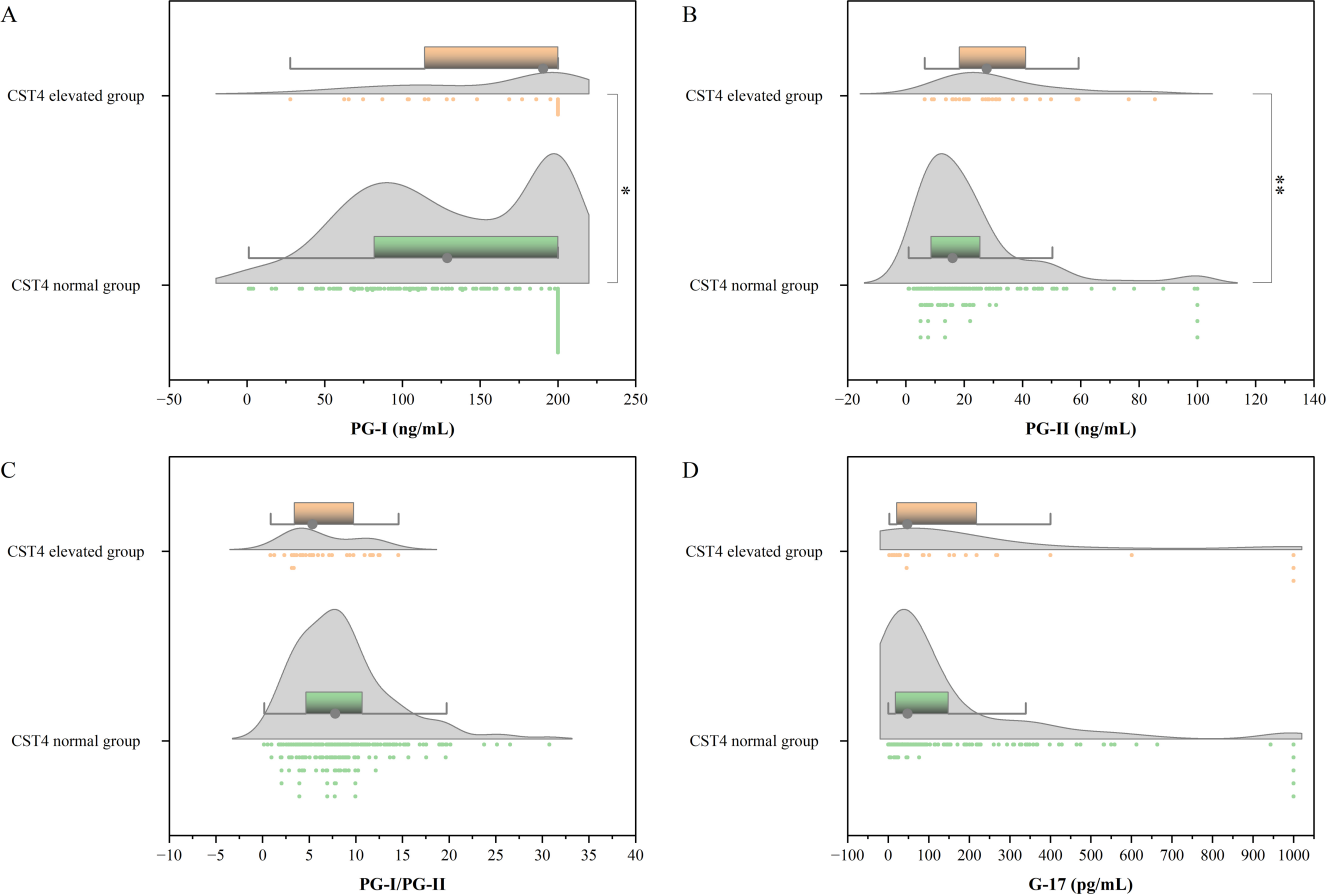
Distribution of gastric function biomarkers according to CST4 status. Raincloud plots illustrate the distributions of **(A)** PG-I, **(B)** PG-II, **(C)** PG-I/PG-II ratio, and **(D)** G-17 levels in patients with elevated CST4 levels and those with normal CST4 levels. Individual data points are shown with overlaid kernel density plots and boxplots indicating median and interquartile ranges. Comparisons between groups were performed using non-parametric tests. *P < 0.05, **P < 0.01.

Stratification by serum CST4 levels revealed significant associations with key gastric biomarkers (**Figure 1**). The CST4-elevated group exhibited significantly higher PG-I (*P* < 0.05) and PG-II (*P* < 0.01) concentrations than the normal group. In contrast, no significant differences were observed in the PG-I/PG-II ratio or G-17 levels between the two groups (*P*> 0.05).

### 3.7 Comparison of serum tumor marker levels between CST4 normal and elevated groups

**Figure 2:**
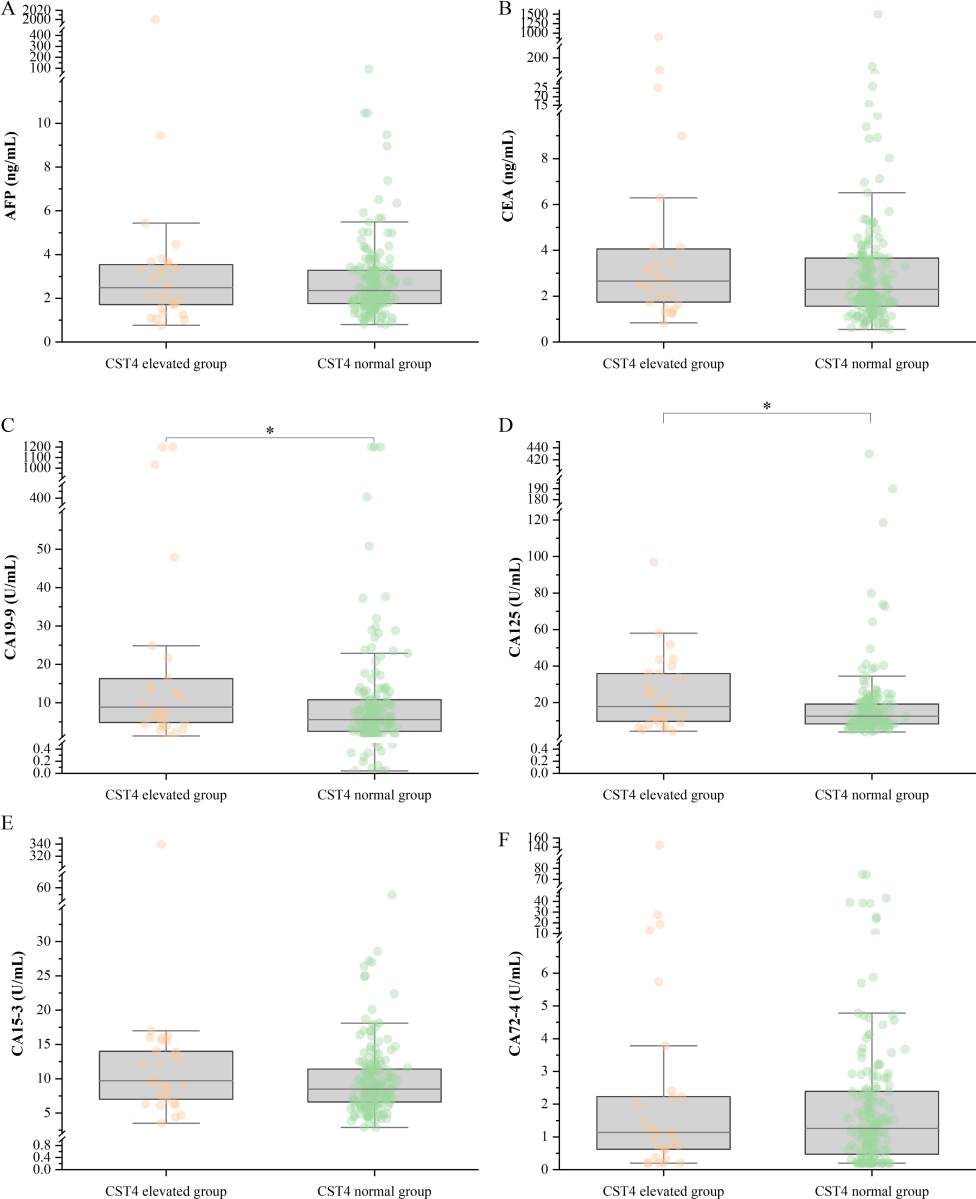
Correlation between CST4 expression and serum tumor marker levels. Box-and-scatter plots show the distributions of serum **(A)** AFP, **(B)** CEA, **(C)** CA19-9, **(D)** CA125, **(E)** CA15-3, and **(F)** CA72–4 in patients with elevated CST4 levels and those with normal CST4 levels. Individual data points are overlaid on boxplots indicating the median and interquartile range. Group comparisons were performed using non-parametric tests. *P < 0.05.

To explore the clinical significance of CST4, serum levels of six common tumor markers (AFP, CEA, CA19-9, CA125, CA15-3, and CA72-4) were compared between CST4-elevated and normal groups (**Figure 2**). The CST4-elevated group exhibited significantly higher serum CA19-9 (*P* < 0.05; **Figure 2C**) and CA125 (*P* < 0.05; **Figure 2D**) concentrations. While median levels of AFP (**Figure 2A**) and CEA (**Figure 2B**) were higher in the CST4-elevated group, these differences were not statistically significant. No significant differences were observed for CA15-3 (**Figure 2E**) or CA72-4 (**Figure 2F**).

### 3.8 Comparison of coagulation function parameters between CST4 normal and elevated groups

**Figure 3:**
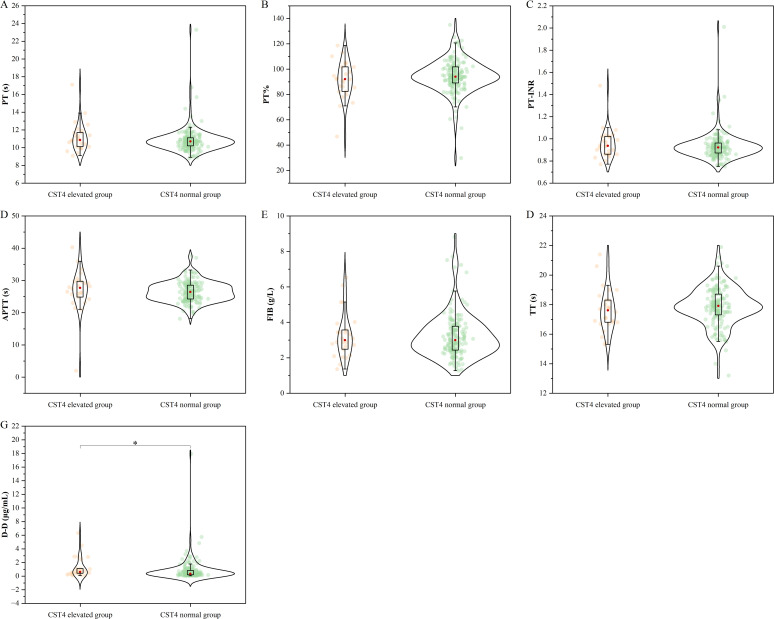
Correlation between CST4 expression and coagulation function. Levels of **(A)** PT, **(B)** PT%, **(C)** PT-INR, **(D)** APTT, **(E)** FIB, **(F)** TT, and **(G)** D-D were compared between the CST4 normal and CST4 elevated groups. Data are presented as violin plots with superimposed box plots. The violin plot shows the probability density of the data, while the box plot indicates the median and interquartile range. *P  < 0.05.

The CST4-elevated group exhibited significantly higher serum DD concentrations (*P* < 0.05; **Figure 3G**). Conversely, other coagulation parameters, including PT, PT%, PT-INR, APTT, FIB, and TT, remained comparable between groups (**Figures 3A–F**).

### 3.9 Impact of *H. pylori* infection and virulence factors on serum biomarker levels

**Figure 4:**
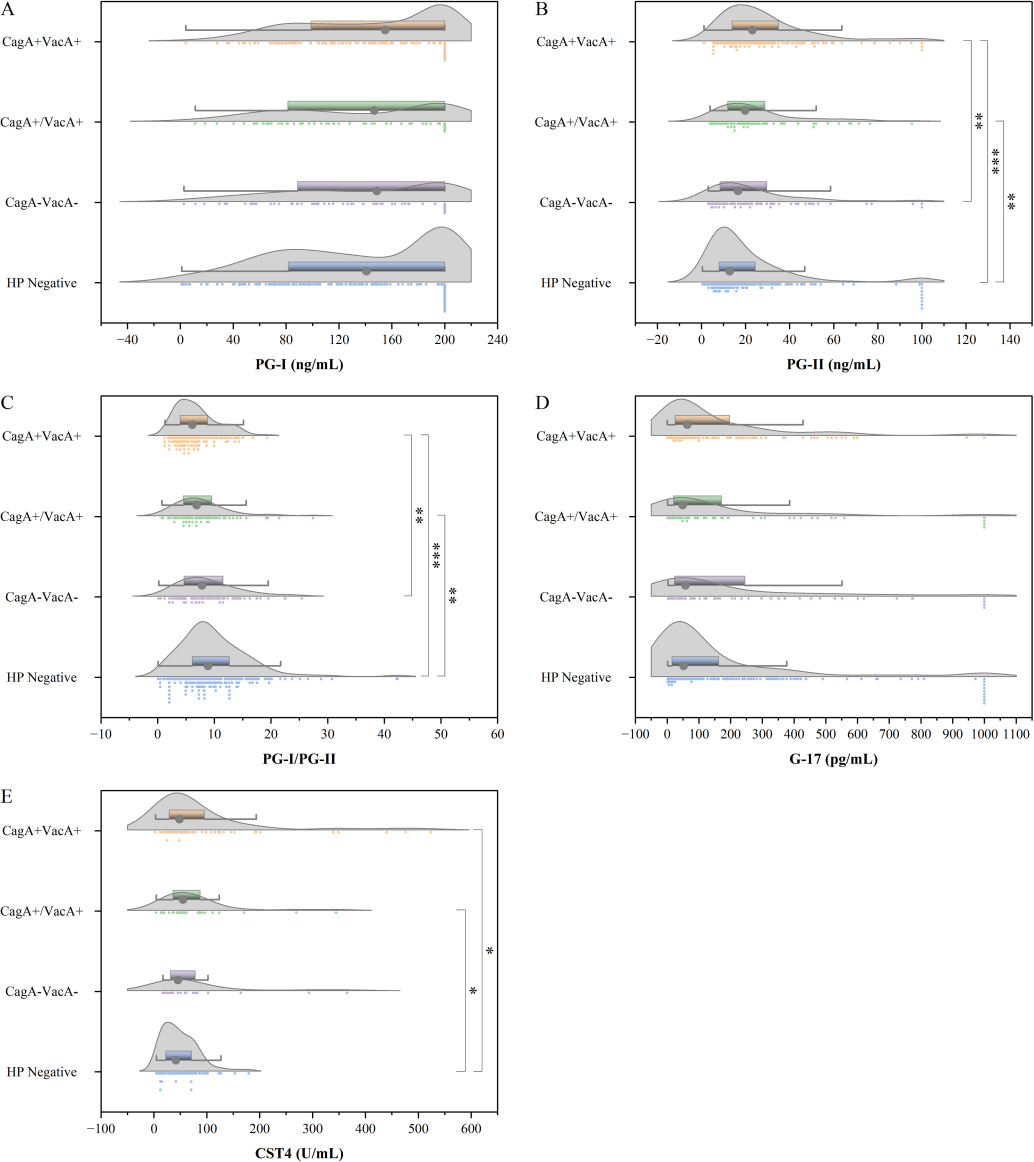
Comparison of serum biomarker levels based on H. pylori infection status and virulence factors. This image displays raincloud plots comparing the serum levels of five biomarkers across four patient groups stratified by H. pylori antibody profiles: HP Negative, CagA-VacA-, CagA+/VacA+ and CagA+VacA+. **(A)** PG-I, **(B)** PG-II, **(C)** PG-I/PG-II, **(D)** G-17, and **(E)** CST4. Individual data points are overlaid with kernel density plots and boxplots indicating the median and interquartile range. Multiple-group comparisons were performed using non-parametric tests with *post hoc* analyses. *P < 0.05, **P < 0.01, ***P  < 0.001.

Hp I-positive groups exhibited significantly higher serum PG-II concentrations than the Hp-negative group (*P* < 0.001 for CagA^+^VacA^+^; *P* < 0.01 for CagA^+^/VacA^+^; **Figure 4B**). The CagA^+^VacA^+^ subtype also showed significantly elevated PG-II relative to the CagA^-^VacA^-^ group (*P* < 0.01). Consequently, the PG-I/PG-II ratio-a surrogate for gastric atrophy-was significantly lower in both Hp I-positive subgroups than in the Hp-negative group (*P* < 0.001 for CagA^+^VacA^+^; *P* < 0.01 for CagA^+^/VacA^+^; **Figure 4C**). The CagA^+^VacA^+^ subgroup exhibited significantly lower PG-I/PG-II ratio compared to the CagA^-^VacA^-^ subgroup (**Figure 4C**, *P* < 0.01).

Serum CST4 levels were also significantly associated with Hp status; both CagA^+^VacA^+^ and CagA^+^/VacA^+^ subtypes exhibited elevated CST4 concentrations compared to the Hp-negative group (*P* < 0.05; **Figure 4E**). The CagA^+^VacA^+^ (*P* < 0.05) and CagA^+^/VacA^+^ (*P* < 0.05) subgroups both exhibited significantly elevated CST4 levels compared to the HP-negative group (**Figure 4E**). Conversely, PG-I (**Figure 4A**) and Gastrin-17 (**Figure 4D**) levels remained comparable among the four groups, despite an upward trend in PG-I within all Hp-positive cohorts.

### 3.10 Association of blood routine parameters with *H. pylori* serotypes

**Figure 5:**
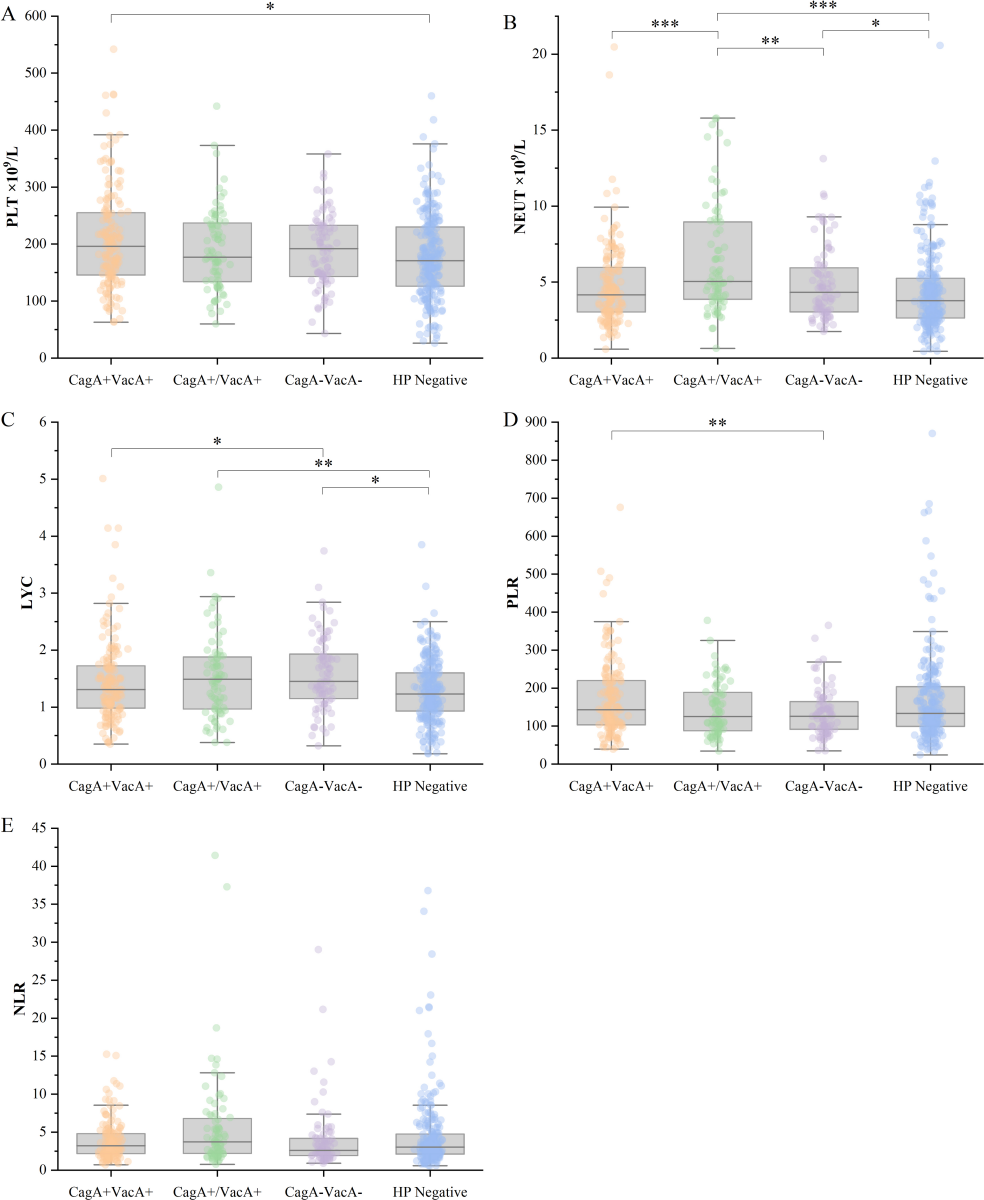
Comparison of blood routine parameters across H. pylori serological groups. The figure displays boxplots comparing hematological parameters across four patient groups stratified by H. pylori serological status: CagA+VacA+, CagA+/VacA+, CagA-VacA-, and HP Negative. **(A)** PLT Count, **(B)** NEUT Count, **(C)** LYC Count, **(D)** PLR, **(E)** NLR. Bars represent mean values with standard deviation, and individual data points are shown. Statistical comparisons were performed using non-parametric tests with *post hoc* multiple comparisons. *P < 0.05, **P < 0.01, ***P < 0.001.

The double-positive CagA^+^VacA^+^ group exhibited significantly higher median PLT counts compared to the Hp-negative group (*P* < 0.05; **Figure 5A**). Regarding systemic inflammation markers, NEUT levels in the single-positive (CagA^+^/VacA^+^) group were significantly higher than in both the CagA^-^VacA^-^ group (*P* < 0.01) and Hp-negative group (*P* < 0.001; **Figure 5B**). Notably, the single-positive group also showed significantly elevated NEUT counts compared to the double-positive CagA^+^VacA^+^ group (*P* < 0.001). Furthermore, the CagA^-^VacA^-^ and single-positive groups exhibited higher median LYC counts than Hp-negative group (*P* < 0.05 and *P* < 0.01, respectively; **Figure 5C**). A significantly higher PLR was observed in the double-positive CagA^+^VacA^+^ group compared with the CagA^-^VacA^-^ group (*P* < 0.01; **Figure 5D**).

### 3.11 Association of serum lipid parameters with *H. pylori* serotypes

**Figure 6:**
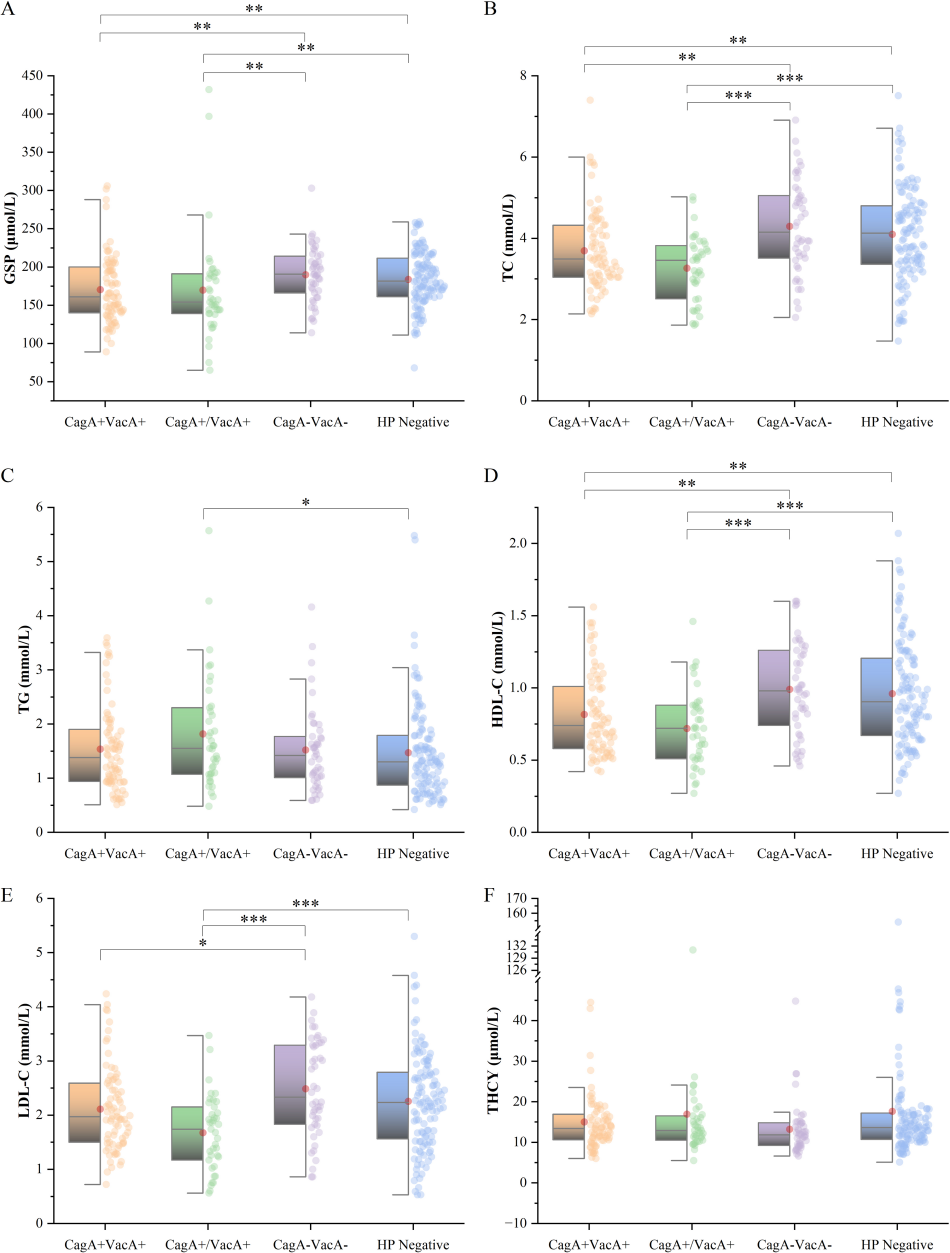
Metabolic and lipid profiles according to Helicobacter pylori virulence factor–defined serotypes. Box-and-scatter plots show the distributions of **(A)** GSP, **(B)** TC, **(C)** TG, **(D)** HDL-C, **(E)** LDL-C, and **(F)** THCY among patients with CagA+VacA+, CagA+/VacA+, CagA-VacA-, and HP Negative status. Boxes indicate the median and interquartile range, and individual data points are overlaid. Statistical comparisons were performed using non-parametric tests with *post hoc* multiple comparisons. *P < 0.05, **P < 0.01, ***P < 0.001.

Hp-negative and CagA^-^VacA^-^ individuals exhibited significantly higher GSP levels compared with both CagA-positive subtypes (double-positive and single-positive; all *P* < 0.01; **Figure 6A**), with no significant difference found between the two CagA-positive groups. Regarding lipid profiles, both CagA-positive groups demonstrated significantly lower concentrations of TC and HDL-C compared to both Hp-negative group and the CagA^-^VacA^-^ group (all *P* < 0.01 or *P* < 0.001; **Figures 6B, D**). Similarly, higher LDL-C concentrations were observed in the Hp-negative and CagA^-^VacA^-^ groups compared with both CagA-positive subtypes, particularly the single-positive (CagA^+^/VacA^+^) group (*P* < 0.001; **Figure 6E**). No significant differences in TG (**Figure 6C**) or THCY (**Figure 6F**) levels were detected among the four groups.

### 3.12 Assessment of hepato-renal function markers in relation to *H. pylori* infection status

**Figure 7:**
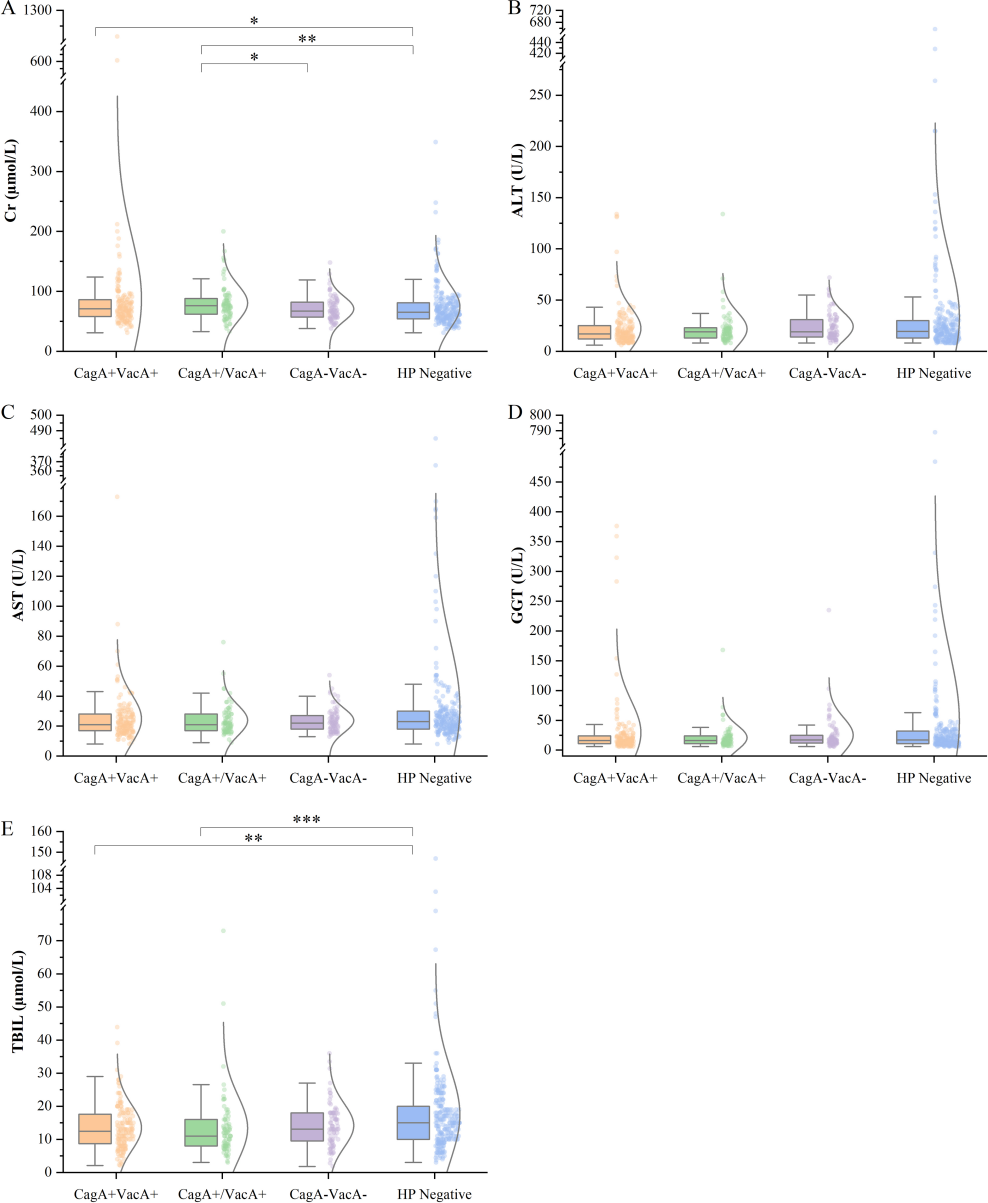
Comparison of hepato-renal function markers based on H. pylori infection status and virulence factors. Serum concentrations of **(A)** Cr, **(B)** ALT, **(C)** AST, **(D)** GGT, and **(E)**TBIL were compared across four patient groups: CagA+VacA+, CagA+/VacA+, CagA-VacA-, and HP Negative. Data are presented as raincloud plots, illustrating the data distribution (density plot), median and interquartile range (box plot), and individual data points. *P < 0.05, **P < 0.01, ***P < 0.001.

Patients infected with CagA-positive strains exhibited higher Cr levels compared to Hp-negative group. Specifically, the single-positive (CagA^+^/VacA^+^) group had significantly higher Cr concentrations than both the CagA^-^VacA^-^ group (*P* < 0.05) and Hp-negative group (*P* < 0.01). Similarly, the double-positive CagA^+^VacA^+^ group showed significantly higher Cr levels than the Hp-negative group (*P* < 0.05; **Figure 7A**). In terms of hepatic markers, both the double-positive (*P* < 0.01) and single-positive (*P* < 0.001) groups exhibited significantly lower TBIL levels compared to the Hp-negative group (**Figure 7E**). Conversely, common liver enzymes-including ALT (**Figure 7B**), AST (**Figure 7C**), and GGT (**Figure 7D**) remained comparable among the four groups (*P* > 0.05).

### 3.13 Coagulation profile alterations in relation to *H. pylori* virulence factors

**Figure 8:**
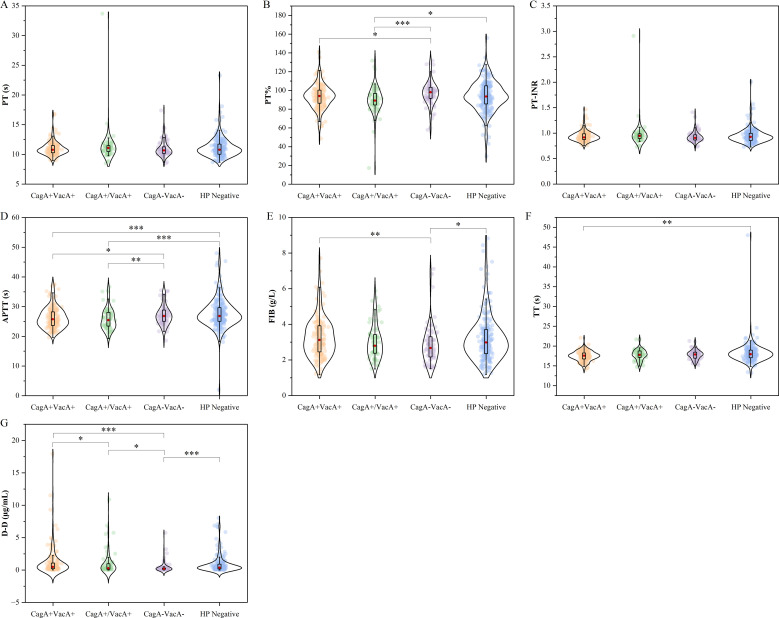
Correlation between H. pylori infection status and coagulation function. Levels of **(A)** PT, **(B)** PT%, **(C)** PT-INR, **(D)** APTT, **(E)** FIB, **(F)** TT, and **(G)** D-D were compared across four patient groups: CagA+VacA+, CagA+/VacA+, CagA-VacA-, and HP Negative. Data are presented as raincloud plots, showing the data distribution (density plot), median and interquartile range (box plot), and individual data points. *P < 0.05, **P < 0.01, ***P  < 0.001.

Pro-coagulant activity was notably higher in CagA-positive cohorts. The PT% was significantly lower in the single-positive (CagA^+^/VacA^+^) group compared to the CagA^-^VacA^-^ (*P* < 0.001) and Hp-negative (*P* < 0.05) groups; a similar reduction was observed in the double-positive CagA^+^VacA^+^ group (*P* < 0.05; **Figure 8B**). No significant differences were found for PT or PT-INR (**Figures 8A, C**). Clotting times were significantly shorter in CagA-positive groups, with the double-positive group showing reduced APTT compared to the CagA^-^VacA^-^ (*P* < 0.05) and Hp-negative (*P* < 0.001) groups. The single-positive group also exhibited shorter APTT values than the CagA^-^VacA^-^ (*P* < 0.01) and Hp-negative groups (*P* < 0.001; **Figure 8D**). Regarding fibrinolytic activity, the CagA^-^VacA^-^ group showed the lowest DD levels, significantly lower than those in the double-positive (*P* < 0.001), single-positive (*P* < 0.05), and Hp-negative (*P* < 0.001) groups. Notably, the double-positive CagA^+^VacA^+^ group exhibited significantly higher DD levels than the single-positive group (*P* < 0.05; **Figure 8G**).

### 3.14 Logistic regression analysis

Multivariate logistic regression was performed to identify independent predictors of Hp infection. The analysis revealed that younger age (OR = 0.976, 95% CI: 0.962-0.991, *P* = 0.001) and a lower PG-I/PG-II ratio (OR = 0.890, 95% CI: 0.846-0.935, *P* < 0.001) were independent predictors of Hp I infection (**Supplementary Figure 1A**). Conversely, no variables demonstrated a statistically significant association with Hp II infection in this model (**Supplementary Figure 1B**).

### 3.15 Development and validation of the prediction nomogram

**Figure 9:**
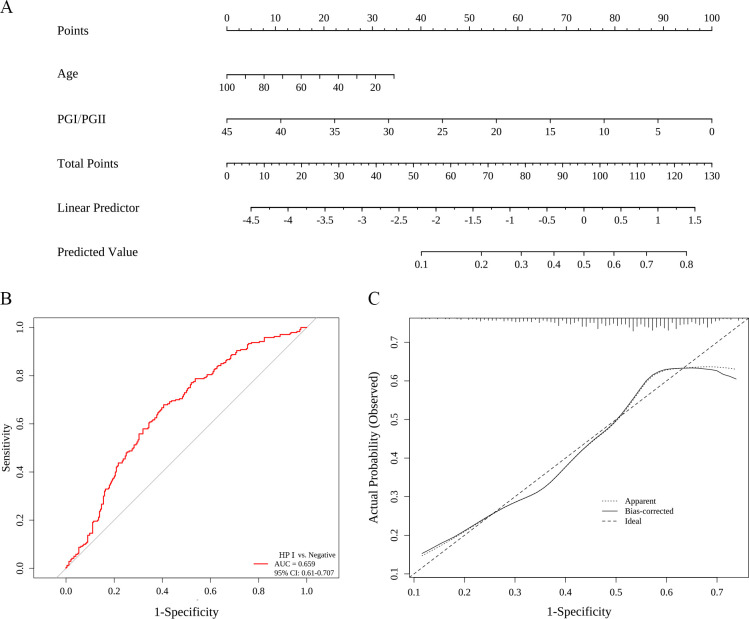
Development and validation of a predictive nomogram for Hp I infection. **(A)** The nomogram integrating Age and PG-I/PG-II ratio to predict the risk probability. **(B)** The ROC curve of the nomogram. **(C)** Calibration curve of the line graph model for predicting the risk of Hp I infection.

A nomogram for predicting the probability of Hp I infection was constructed based on age and the PG-I/PG-II ratio (**Figure 9A**). The model demonstrated moderate discrimination, yielding an AUC of 0.659 (**Figure 9B**). Calibration was satisfactory, as indicated by the close agreement between predicted and observed probabilities (**Figure 9C**). Standardized decision curve analysis (sDCA) further confirmed the clinical utility of the model across a threshold range of 0-0.75, showing a superior standardized net benefit compared to the ‘treat-all’ strategy (**Supplementary Figure 2**).

### 3.16 Independent predictors identified by multivariate analysis

Age was inversely associated with polyp risk (OR = 0.954, 95% CI: 0.927–0.982, *P* = 0.001). G17 was inversely associated with polyp risk (OR = 0.998, 95% CI: 0.997-1.000, *P* = 0.033), while serum TBIL levels also demonstrated an inverse relationship with polyp presence (OR = 0.941, 95% CI: 0.908–0.975, *P* < 0.001). Additionally, PLR was negatively associated with polyp presence (OR = 0.996, 95% CI: 0.993–0.999, *P* = 0.021) (**Supplementary Figure 3A**). Age was inversely associated with ulcer risk (OR = 0.966, 95% CI: 0.939–0.993, *P*= 0.015). Among coagulation metrics, PT-INR exhibited a robust inverse association with ulcer risk (OR = 0.009, 95% CI: 0.000–0.274, *P* = 0.007), whereas APTT was positively associated with the outcome (OR = 1.130, 95% CI: 1.027–1.244, *P* = 0.013; **Supplementary Figure 3B**). The PG-I/PG-II ratio was significantly inversely associated with malignancy (OR = 0.875, 95% CI: 0.812–0.942, *P* < 0.001). TT also showed a negative association (OR = 0.672, 95% CI: 0.490–0.921, *P* = 0.014) (**Supplementary Figure 3C**).

### 3.17 Development and validation of a predictive nomogram

**Figure 10:**
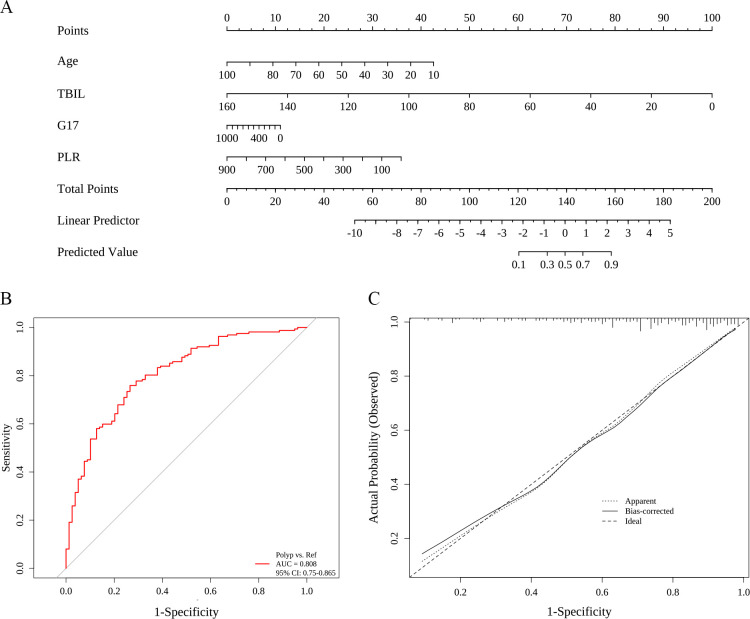
Nomogram for predicting gastric polyp risk and its validation. **(A)** Clinical prediction nomogram incorporating Age, TBIL, G17, and PLR. **(B)** ROC curve illustrating the model's discriminative ability. **(C)** Calibration plot comparing the nomogram's predicted probabilities with the actual observed probabilities.

**Figure 11:**
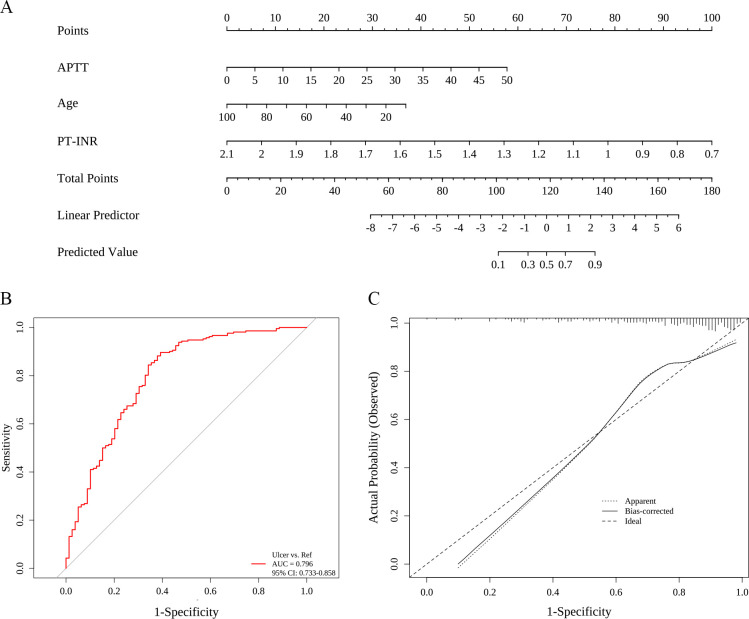
Nomogram for predicting gastric ulcer risk and its validation. **(A)** Nomogram for predicting gastric ulcer risk, incorporating the variables Age, APTT, and PT-INR. **(B)** ROC curve assessing model discrimination. **(C)** Calibration plot evaluating the agreement between predicted and observed event rates.

A predictive nomogram for gastric polyps was constructed incorporating four independent predictors: age, TBIL, G-17, and PLR (**Figure 10A**). The model demonstrated strong discriminative power with an AUC of 0.82 (**Figure 10B**). The calibration curve showed high consistency between predicted and observed outcomes, closely aligning with the ideal 45-degree line (**Figure 10C**). For gastric ulcer risk, a second nomogram was developed using age, APTT, and PT-INR as predictors (**Figure 11A**). This model yielded an AUC of 0.85, indicating excellent predictive accuracy (**Figure 11B**). The corresponding calibration plot confirmed close agreement between the predicted and observed ulcer probabilities (**Figure 11C**).

**Figure 12:**
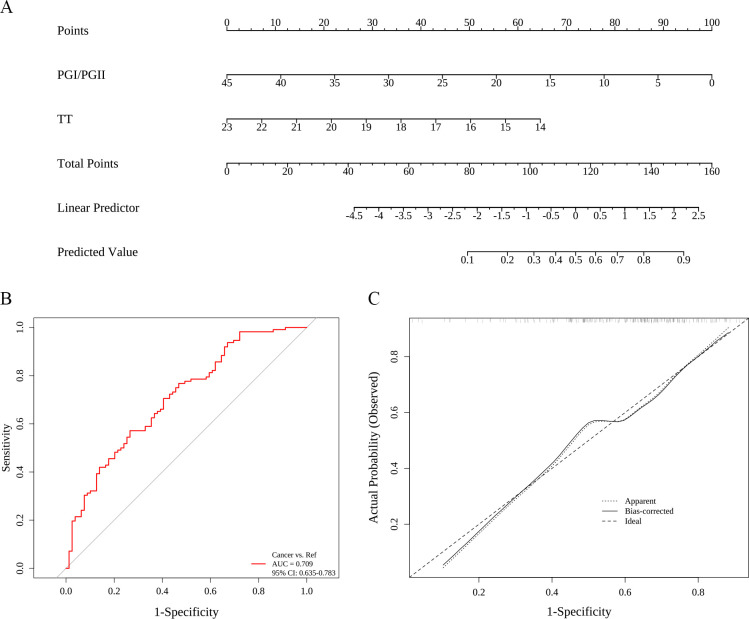
Nomogram for predicting gastric cancer risk and its validation. **(A)** Clinical prediction nomogram integrating PG-I/PG-II and TT. **(B)** ROC curve assessing the model's discrimination performance. **(C)** Calibration plot showing predicted versus observed cancer probabilities.

Finally, a nomogram for gastric cancer risk was established based on the PGI/II ratio and TT (**Figure 12A**). The model’s discriminative performance was robust, with an ROC curve yielding an AUC of 0.78 (**Figure 12B**). Calibration remained accurate across the entire range of predicted probabilities (**Figure 12C**). sDCA demonstrated that all three gastric lesion models (polyp, ulcer, and cancer) provided a superior standardized net benefit compared to the ‘treat-all’ or ‘treat-none’ strategies across most threshold probabilities. Notably, the cancer model was optimal at lower-to-intermediate risk thresholds, whereas the ulcer model excelled at higher thresholds, supporting their application in risk-stratified clinical management (**Supplementary Figure 4**).

## 4 Discussion

This retrospective study comprehensively analyzes the systemic serological alterations associated with *H. pylori* infection, focusing on virulent CagA-positive strains and the novel biomarker CST4. Beyond confirming the established link between CagA-positive Hp and severe gastric pathology, we reveal a distinct broad systemic ‘serological signature’. Notably, we identified a significant association between elevated serum CST4 levels and CagA-positive infection. While CST4 did not emerge as an independent predictor in the disease-specific models, its elevation reflects a broad systemic response to high-virulence strains, highlighting its role as an exploratory pathophysiological indicator.

Our results confirm that CagA-positive Hp infection, particularly the CagA^+^VacA^+^ subtype, is strongly associated with peptic ulcer disease and gastric mucosal damage. The significantly elevated PG-II levels and decreased PGI/II ratio in CagA-positive groups align with the established pathophysiology: CagA disrupts epithelial cell polarity and signaling, thereby augmenting inflammation and atrophic changes (Hatakeyama, 2006; Shiota et al., 2014; Baj et al., 2020). The PGI/II ratio remains a gold-standard non-invasive surrogate for gastric atrophy within the Correa cascade (Pimentel-Nunes et al., 2019; Malfertheiner et al., 2022). Its nadir in CagA-positive groups, including malignancy cases, underscores the role of virulent strains in driving oncogenesis, consistent with evidence identifying CagA as a critical driver of gastric cancer progression (Hatakeyama, 2006; Pormohammad et al., 2018).

A central contribution of this study is the characterization of a systemic serological ‘fingerprint’ unique to CagA-positive Hp infection. We observed significant alterations across multiple physiological axes. While previous literature frequently reports elevated NLR in severe *H. pylori* infection as a systemic inflammation marker (Farah and Khamisy-Farah, 2014), NLR did not differ significantly across our virulence subgroups (*P* = 0.298). This aligns with studies indicating that systemic leukocyte responses (e.g., NLR elevation) are primarily driven by macroscopic gastric mucosal damage (e.g., ulcers) rather than bacterial CagA status independently (Jafarzadeh et al., 2013). Therefore, while CagA-positive strains may not overtly skew systemic leukocyte ratios in the absence of severe local lesions, they nevertheless provoke a state of chronic, low-grade systemic stress. This is evidenced by the stimulated release of pro-inflammatory cytokines (e.g., IL-1β, IL-6, and TNF-α) (de Korwin et al., 2017; Xie et al., 2023; Yu et al., 2023), as well as a distinct hypolipidemic profile characterized by significantly lower TC, HDL-C, and LDL-C. This suggests that these strains uniquely perturb lipid homeostasis, likely through inflammatory-mediated suppression of hepatic lipid synthesis or enhanced clearance during the acute-phase response (Khovidhunkit et al., 2004). Importantly, we acknowledge that markers such as lipid profiles and inflammatory indices are non-specific acute-phase reactants. Rather than indicating a direct, targeted disruption of specific organ systems by *H. pylori*, these alterations more likely reflect a generalized, downstream systemic inflammatory burden induced by chronic virulent infection.

The observed shortening of APTT and elevation of DD in these groups further point toward a systemic hypercoagulable state. Chronic Hp infection can provoke platelet activation and foster a pro-thrombotic endothelial phenotype, providing a mechanistic link to the increased cardiovascular risk associated with CagA-positive strains (de Korwin et al., 2017; Santos et al., 2020). Additionally, elevated serum creatinine levels in CagA-positive groups suggest subclinical renal impairment, potentially mediated by immune complex deposition or chronic inflammatory stress (Pan et al., 2019; Liu et al., 2020). Collectively, this systemic profile reinforces the paradigm of Hp as a systemic pathogen modulating host physiology far beyond the gastric niche (Gravina et al., 2018; Santos et al., 2020).

A novel finding is the significant association between elevated serum CST4 levels and CagA-positive infection. CST4, a cysteine protease inhibitor, is a known modulator of extracellular matrix (ECM) remodeling, inflammation, and tumor invasion (Leto et al., 2018; Gu et al., 2025). While its role in gastric malignancy is emerging (Gu et al., 2025), its specific induction by virulent Hp strains has not been previously detailed.

Our data suggest that CST4 is upregulated as a host counter-regulatory response to the intense inflammatory and proteolytic stimuli triggered by CagA-positive strains. Notably, its correlation with markers of gastric activity (PG-I/II), tumor progression (CA19-9/125), and coagulation (D-dimer) highlights its role as an indicator of a systemic pro-tumorigenic and pro-thrombotic milieu. Although CST4 was not identified as an independent predictor in the final multivariable models—likely due to high collinearity with CagA status—it remains a valuable exploratory marker for screening high-virulence infection and monitoring the systemic host response (Gu et al., 2025; Koopaie et al., 2025).

Integrating CST4 into pre-endoscopic screening algorithms, combined with serological virulence typing, represents a promising research direction.

The nomograms developed for gastric polyps, ulcers, and cancer represent a pragmatic translation of our findings into clinical practice. By integrating readily accessible parameters—such as age, the PGI/PGII ratio, and coagulation markers—these models achieved robust discriminative performance. Crucially, sDCA demonstrated superior net benefits compared to a ‘treat-all’ strategy across a broad range of threshold probabilities. This utility is vital for risk-stratified management, enabling clinicians to prioritize high-risk individuals for intensive endoscopic surveillance while avoiding unnecessary interventions in low-risk patients (Vickers and Elkin, 2006; Cai et al., 2019). Our approach aligns with the shift toward multi-parameter modeling in Hp-related disease assessment, moving beyond the limitations of single-biomarker strategies (Yamaguchi et al., 2016; Cai et al., 2019).

A final consideration pertains to the benchmarking of our clinical models. It is important to contextualize the utility of our proposed nomograms. For gastric cancer risk assessment, established systems such as the OLGA/OLGIM stages and the “ABC method” are widely recognized. However, a direct head-to-head statistical comparison was hindered by the retrospective nature of our study, as OLGA staging requires specific multi-point biopsy mapping which is not uniformly available in real-world clinical records (Yamaguchi et al., 2016; Rugge et al., 2018; Cai et al., 2019). Furthermore, while the ABC method provides broad ordinal risk categorization, our serology-based nomograms offer a continuous, individualized probability score by integrating extra-gastric systemic parameters. Most importantly, while validated benchmark models exist predominantly for malignancy, there is a distinct lack of universally accepted, serology-based predictive nomograms for benign but clinically burdensome lesions like gastric polyps and peptic ulcers (Yamaguchi et al., 2016; Cai et al., 2019). Thus, our models uniquely address this critical gap by providing a comprehensive, non-invasive triage tool across the spectrum of *H. pylori*-associated diseases.

The strengths of this study include its robust sample size, systemic serological mapping, virulence stratification, and validated clinical decision tools. However, several limitations warrant consideration. First, the retrospective, single-center design confined to a specific geographic population in China introduces inherent selection bias and limits the generalizability of our findings and the proposed markers to other regions or ethnicities. Second, the attrition of CST4 data necessitates conservative interpretation of its diagnostic performance. Third, while indispensable for identifying specific virulence factors (e.g., CagA, VacA), IgG immunoblotting reflects host immune memory rather than active bacterial load, limiting the confirmation of concurrent active infection (Patel et al., 2014). Future studies must integrate virulence serotyping with active infection markers (e.g., UBT) and gastric functional indices (e.g., pepsinogens) to establish a definitive classification. Fourth, the reference group was defined by the absence of gastric lesions, rather than strict Hp negativity; 51.9% were Hp-positive (35.4% Hp I, 16.5% Hp II), potentially attenuating observed differences and yielding conservative comparisons. Finally, our cross-sectional framework precludes establishing definitive causal relationships between Hp virulence factors and the observed broad systemic alterations.

Future prospective, multicenter cohorts are required to validate these findings and elucidate causal dynamics. Specifically, evaluating serum CST4 levels dynamically—both pre- and post-Hp eradication—will clarify its role as a functional response or prognostic biomarker (Gu et al., 2025). Integrating gastric mucosal histology, such as OLGA/OLGIM staging, with high-resolution bacterial genomic analysis (e.g., CagA EPIYA motifs and VacA s/m regions) will provide deeper mechanistic insights into genotype-driven systemic alterations (Wen and Moss, 2009; Magister and Kos, 2013). Furthermore, exploring the functional role of CST4 in Hp-infected gastric epithelium via *in vitro*/*in vivo* models could reveal the precise tissue remodeling pathways (Magister and Kos, 2013). Investigating whether Hp eradication reverses the identified systemic abnormalities (e.g., hypolipidemia and hypercoagulability) will be crucial for assessing the long-term clinical relevance of these extra-gastric effects (de Luis et al., 1999; Torgano et al., 1999; Yusuf and Mishra, 2002; Watanabe et al., 2021). Ultimately, the management of Hp infection is shifting toward personalized, virulence-informed strategies (Malfertheiner et al., 2022; Suresh et al., 2025). Our nomograms provide a robust framework for this paradigm, potentially optimizing risk-stratified screening and eradication policies, particularly in high-incidence regions (Liou et al., 2020; Thrift et al., 2023).

In summary, this study establishes that CagA-positive Helicobacter pylori infection triggers a distinct and adverse systemic serological signature. These alterations—spanning inflammatory, metabolic, coagulation, and renal axes—demonstrate that the pathological impact of virulent Hp strains extends far beyond the gastric mucosa. Furthermore, while its strong collinearity with CagA precludes its utility as an independent predictor, serum CST4 still emerges as an exploratory pathophysiological indicator reflecting downstream host systemic responses to virulence. Finally, our validated nomograms for predicting gastric polyps, ulcers, and malignancy offer robust, data-driven tools for clinicians. By integrating bacterial virulence with host serological profiles, these models facilitate an individualized, risk-stratified management, potentially optimizing healthcare resources and improving outcomes in populations with high Hp prevalence.

## Data Availability

The original contributions presented in the study are included in the article/**Supplementary Material**. Further inquiries can be directed to the corresponding authors.
