# Supplementary material for: Systemic serological signatures and novel biomarker CST4 link CagA-positive Helicobacter pylori to broad systemic alterations
Source: Front Cell Infect Microbiol. 2026 Jun 26;16:1826407. doi: 10.3389/fcimb.2026.1826407 (PMC13350065; doi:10.3389/fcimb.2026.1826407)
Supplement: Supplementary file 1 [file Table1.docx]

**Supplementary Table 1.**Distribution of *H. pylori* Antibody Profiles in Different Groups

| **Group** | **HpⅠ**  **(n=240)** | | | | | | | | | | | |  | **HpⅡ**  **(n=81)** | | |
| --- | --- | --- | --- | --- | --- | --- | --- | --- | --- | --- | --- | --- | --- | --- | --- | --- |
| **Subgroup** | **CagA^+^VacA^+^**  **(n=160)** | | | | **CagA^+^/VacA^+^**  **(n=80)** | | | | | | | |  | **CagA^-^VacA^-^** | | |
| **Hp antibody typing** | CagA^+^VacA^+^UreA^+^UreB^+^ | CagA^+^VacA^+^UreA^+^UreB^-^ | CagA^+^VacA^+^UreA^-^UreB^+^ | CagA^+^VacA^+^UreA^-^UreB^-^ | CagA^+^VacA^-^UreA^+^UreB^+^ | CagA^+^VacA^-^UreA^+^UreB^-^ | CagA^+^VacA^-^UreA^-^UreB^+^ | CagA^+^VacA^-^UreA^-^UreB^-^ | CagA^-^VacA^+^UreA^+^UreB^+^ | CagA^-^VacA^+^UreA^+^UreB^-^ | CagA^-^VacA^+^UreA^-^UreB^+^ | CagA^-^VacA^+^UreA^-^UreB^-^ |  | CagA^-^VacA^-^UreA^+^UreB^+^ | CagA^-^VacA^-^UreA^+^UreB^-^ | CagA^-^VacA^-^UreA^-^UreB^+^ |
| **No. (%)** | 141  (58.75) | 0  (0.00) | 19  (7.92) | 0  (0.00) | 24  (10.00) | 0  (0.00) | 38  (15.83) | 14  (5.83) | 3  (1.25) | 0  (0.00) | 1  (0.42) | 0  (0.00) |  | 19  (23.46) | 0  (0.00) | 62  (76.54) |

**Supplementary Table 2.** Distribution of *H. pylori* Antibody Profiles Across Different Clinical Groups

| **Group** | **Hp antibody typing**  **No. (%)** | **Overall**  **(n=565)** | **Control group**  **(n=79)** | **CG/Polyp**  **(n=212)** | **Ulcer**  **(n=162)** | **GC/CRC/EC**  **(n=112)** | ***χ^2^*** | ***P*** |
| --- | --- | --- | --- | --- | --- | --- | --- | --- |
| **HpⅠ**  **(n=240)** | **CagA^+^VacA^+^** | 160 (28.32) | 16 (20.25) | 44 (20.75) | 63 (38.89) | 37 (33.04) | 18.651 | 0.000 |
|  | CagA^+^VacA^+^UreA^+^UreB^+^ | 141 (24.96) | 13 (16.46) | 39 (18.40) | 57 (35.19) | 32 (28.57) | / | / |
|  | CagA^+^VacA^+^UreA^-^UreB^+^ | 19 (3.36) | 3 (3.80) | 5 (2.36) | 6 (3.70) | 5 (4.46) | / | / |
|  | **CagA^+^/VacA^+^** | 80 (14.16) | 12 (15.19) | 24 (11.32) | 26 (16.05) | 18 (16.07) | 2.287 | 0.515 |
|  | CagA^+^VacA^-^UreA^+^UreB^+^ | 24 (4.25) | 2 (2.53) | 6 (2.83) | 10 (6.17) | 6 (5.36) | / | / |
|  | CagA^+^VacA^-^UreA^-^UreB^+^ | 38 (6.73) | 8 (10.13) | 12 (5.66) | 14 (8.64) | 4 (3.57) | / | / |
|  | CagA^+^VacA^-^UreA^-^UreB^-^ | 14 (2.48) | 2 (2.53) | 4 (1.89) | 2 (1.23) | 6 (5.36) | / | / |
|  | CagA^-^VacA^+^UreA^+^UreB^+^ | 3 (0.53) | 0 (0.00) | 1 (0.47) | 0 (0.00) | 2 (1.79) | / | / |
|  | CagA^-^VacA^+^UreA^-^UreB^+^ | 1 (0.18) | 0 (0.00) | 1 (0.47) | 0 (0.00) | 0 (0.00) | / | / |
| **HpⅡ**  **(n=81)** | **CagA^-^VacA^-^** | 81 (14.34) | 13 (16.46) | 29 (13.68) | 25 (15.43) | 14 (12.50) | 0.829 | 0.842 |
|  | CagA^-^VacA^-^UreA^+^UreB^+^ | 19 (3.36) | 3 (3.80) | 2 (0.94) | 10 (6.17) | 4 (3.57) | / | / |
|  | CagA^-^VacA^-^UreA^-^UreB^+^ | 62 (10.97) | 10 (12.66) | 27 (12.74) | 15 (9.26) | 10 (8.93) | / | / |

**Supplementary Table 3.** Association between Patient Variables and Seropositivity of *H. pylori* Virulence Factors

| **Patient Variable** | **CagA (116KD)** | |  | **VacA (91KD)** | |  | **VacA (95KD)** | |  | **UreA (30KD)** | |  | **UreB (66KD)** | |
| --- | --- | --- | --- | --- | --- | --- | --- | --- | --- | --- | --- | --- | --- | --- |
|  | Positive  (n=236) | Negative  (n=329) |  | Positive  (n=164) | Negative  (n=401) |  | Positive  (n=164) | Negative  (n=401) |  | Positive  (n=187) | Negative  (n=378) |  | Positive  (n=307) | Negative  (n=258) |
| Male sex, No. | 158 (66.95) | 188 (57.14) |  | 108 (65.85) | 238 (59.35) |  | 108 (65.85) | 238 (59.35) |  | 128 (68.45) | 218 (57.67) |  | 50 (40.65) | 140 (48.11) |
| *χ^2^* | 5.567 | |  | 2.073 | |  | 2.073 | |  | 6.122 | |  | 5.887 | |
| *P* | 0.018 | |  | 0.150 | |  | 0.150 | |  | 0.013 | |  | 0.015 | |
| Age, median (IQR) | 64.00  (54.00, 73.00) | 67.00  (55.00, 77.00) |  | 60.50  (52.25, 71.75) | 67.00  (55.00, 77.00) |  | 60.50  (52.25, 71.75) | 67.00  (55.00, 77.00) |  | 60.00  (53.00, 73.00) | 67.00  (55.00, 76.25) |  | 67.00  (55.00, 77.00) | 67.00  (55.00, 77.00) |
| *Z* | -2.380 | |  | -2.997 | |  | -2.997 | |  | -2.911 | |  | -2.342 | |
| *P* | 0.017 | |  | 0.003 | |  | 0.003 | |  | 0.004 | |  | 0.019 | |
| <40 | 20 (8.47) | 23 (6.99) |  | 16 (9.76) | 27 (6.73) |  | 16 (9.76) | 27 (6.73) |  | 18 (9.63) | 25 (6.61) |  | 26 (8.47) | 17 (6.59) |
| 40-59 | 84 (35.59) | 104 (31.61) |  | 61 (37.20) | 127 (31.67) |  | 61 (37.20) | 127 (31.67) |  | 72 (38.50) | 116 (30.69) |  | 112 (36.48) | 76 (29.46) |
| ≥60 | 132 (55.93) | 202 (61.40) |  | 87 (53.05) | 247 (61.60) |  | 87 (53.05) | 247 (61.60) |  | 97 (51.87) | 237 (62.70) |  | 169 (55.05) | 165 (63.95) |
| *χ^2^* | 1.747 | |  | 3.904 | |  | 3.904 | |  | 6.268 | |  | 4.610 | |
| *P* | 0.417 | |  | 0.142 | |  | 0.142 | |  | 0.044 | |  | 0.100 | |
| BMI(n=425), (mean ±SD) | 23.21±4.19 | 22.62±3.83 |  | 23.16±4.24 | 22.73±3.87 |  | 23.16±4.24 | 22.73±3.87 |  | 23.49±4.25 | 22.54±3.81 |  | 23.15±4.19 | 22.54±3.73 |
| ***t*** | -1.501 | |  | -1.034 | |  | -1.034 | |  | -2.356 | |  | -1.588 | |
| *P* | 0.134 | |  | 0.302 | |  | 0.302 | |  | 0.019 | |  | 0.113 | |
| Underweight (BMI < 18.5), No. (%) | 19 (10.80) | 35 (14.06) |  | 16 (12.21) | 38 (12.93) |  | 16 (12.21) | 38 (12.93) |  | 16 (11.03) | 38 (13.57) |  | 27 (12.05) | 27 (13.43) |
| Normal (18.5 ≤ BMI < 25.0), No. (%) | 104 (59.09) | 151 (60.64) |  | 76 (58.02) | 179 (60.88) |  | 76 (58.02) | 179 (60.88) |  | 82 (56.55) | 173 (61.79) |  | 129 (57.59) | 126 (62.69) |
| Overweight (25.0 ≤ BMI < 30.0), No. (%) | 45 (25.57) | 57 (22.89) |  | 33 (25.19) | 69 (23.47) |  | 33 (25.19) | 69 (23.47) |  | 38 (26.21) | 64 (22.86) |  | 59 (26.34) | 43 (21.39) |
| Obese (BMI ≥ 30.0), No. (%) | 8 (4.55) | 6 (2.41) |  | 6 (4.58) | 8 (2.72) |  | 6 (4.58) | 8 (2.72) |  | 9 (6.21) | 5 (1.79) |  | 9 (4.02) | 5 (2.49) |
| *χ^2^* | 2.640 | |  | 1.223 | |  | 1.223 | |  | 7.035 | |  | 2.450 | |
| *P* | 0.451 | |  | 0.747 | |  | 0.747 | |  | 0.071 | |  | 0.484 | |
| **Missing, No. (%)** | 60 (25.42) | 80 (24.32) |  | 33 (20.12) | 107 (26.68) |  | 33 (20.12) | 107 (26.68) |  | 42 (22.46) | 98 (25.93) |  | 83 (27.04) | 57 (22.09) |
| **Smoking status, No. (%)** | | | | | | | | | | | | | | |
| Former/current | 74 (31.36) | 58 (17.63) |  | 49 (29.88) | 83 (20.70) |  | 49 (29.88) | 83 (20.70) |  | 54 (28.88) | 78 (20.63) |  | 82 (26.71) | 50 (19.38) |
| Never | 162 (68.64) | 271 (82.37) |  | 115 (70.12) | 318 (79.30) |  | 115 (70.12) | 318 (79.30) |  | 133 (71.12) | 300 (79.37) |  | 225 (73.29) | 208 (80.62) |
| *χ^2^* | 14.462 | |  | 5.478 | |  | 5.478 | |  | 4.747 | |  | 4.207 | |
| *P* | 0.000 | |  | 0.019 | |  | 0.019 | |  | 0.029 | |  | 0.040 | |
| **Drinking status, No. (%)** | | | | | | | | | | | | | | |
| Former/current | 55 (23.31) | 53 (16.11) |  | 37 (22.56) | 71 (17.71) |  | 37 (22.56) | 71 (17.71) |  | 39 (20.86) | 69 (18.25) |  | 64 (20.85) | 44 (17.05) |
| Never | 181 (76.69) | 276 (83.89) |  | 127 (77.44) | 330 (82.29) |  | 127 (77.44) | 330 (82.29) |  | 148 (79.14) | 309 (81.75) |  | 243 (79.15) | 214 (82.95) |
| *χ^2^* | 4.602 | |  | 1.775 | |  | 1.775 | |  | 0.548 | |  | 1.304 | |
| *P* | 0.032 | |  | 0.183 | |  | 0.183 | |  | 0.459 | |  | 0.253 | |
| **Hypertension, No. (%)** | | | | | | | | | | | | | | |
| Yes | 89 (37.71) | 133 (40.43) |  | 57 (34.76) | 165 (41.15) |  | 57 (34.76) | 165 (41.15) |  | 68 (36.36) | 154 (40.74) |  | 119 (38.76) | 103 (39.92) |
| No | 147 (62.29) | 196 (59.57) |  | 107 (65.24) | 236 (58.85) |  | 107 (65.24) | 236 (58.85) |  | 119 (63.64) | 224 (59.26) |  | 188 (61.24) | 155 (60.08) |
| *χ^2^* | 0.424 | |  | 1.993 | |  | 1.993 | |  | 1.005 | |  | 0.079 | |
| *P* | 0.515 | |  | 0.158 | |  | 0.158 | |  | 0.316 | |  | 0.778 | |
| [**Cardiac disease**](file:///C:\Program%20Files\baidu-translate-client\resources\app.asar\app.html)**, No. (%)** | | | | | | | | | | | | | | |
| Yes | 29 (12.29) | 44 (13.37) |  | 19 (11.59) | 54 (13.47) |  | 19 (11.59) | 54 (13.47) |  | 22 (11.76) | 51 (13.49) |  | 41 (13.36) | 32 (12.40) |
| No | 207 (87.71) | 285 (86.63) |  | 145 (88.41) | 347 (86.53) |  | 145 (88.41) | 347 (86.53) |  | 165 (88.24) | 327 (86.51) |  | 266 (86.64) | 226 (87.60) |
| *χ^2^* | 0.144 | |  | 0.366 | |  | 0.366 | |  | 0.332 | |  | 0.113 | |
| *P* | 0.704 | |  | 0.545 | |  | 0.545 | |  | 0.565 | |  | 0.737 | |
| **Diabetes, No. (%)** | | | | | | | | | | | | | | |
| Yes | 31 (13.14) | 37 (11.25) |  | 19 (11.59) | 49 (12.22) |  | 19 (11.59) | 49 (12.22) |  | 23 (12.30) | 45 (11.90) |  | 42 (13.68) | 26 (10.08) |
| No | 205 (86.86) | 292 (88.75) |  | 145 (88.41) | 352 (87.78) |  | 145 (88.41) | 352 (87.78) |  | 164 (87.70) | 333 (88.10) |  | 265 (86.32) | 232 (89.92) |
| *χ^2^* | 0.463 | |  | 0.044 | |  | 0.044 | |  | 0.018 | |  | 1.719 | |
| *P* | 0.496 | |  | 0.833 | |  | 0.833 | |  | 0.892 | |  | 0.190 | |
| **Age-adjusted Charlson comorbidity index, No. (%)** | | | | | | | | | | | | | | |
| 0 | 11 (4.66) | 32 (9.73) |  | 10 (6.10) | 33 (8.23) |  | 10 (6.10) | 33 (8.23) |  | 11 (5.88) | 32 (8.47) |  | 17 (5.54) | 26 (10.08) |
| 1 | 44 (18.64) | 49 (14.89) |  | 33 (20.12) | 60 (14.96) |  | 33 (20.12) | 60 (14.96) |  | 36 (19.25) | 57 (15.08) |  | 58 (18.89) | 35 (13.57) |
| ≥2 | 181 (76.69) | 248 (75.38) |  | 121 (73.78) | 308 (76.81) |  | 121 (73.78) | 308 (76.81) |  | 140 (74.87) | 289 (76.46) |  | 232 (75.57) | 197 (76.36) |
| *χ^2^* | 5.839 | |  | 2.718 | |  | 2.718 | |  | 2.461 | |  | 6.225 | |
| *P* | 0.054 | |  | 0.257 | |  | 0.257 | |  | 0.292 | |  | 0.044 | |
| SBP (mmHg), median (IQR) | 121.50  (107.00, 134.75) | 123.00  (109.00, 137.50) |  | 118.50  (107.00, 132.00) | 124.00  (109.00, 138.00) |  | 118.50  (107.00, 132.00) | 124.00  (109.00, 138.00) |  | 118.00  (107.00, 132.00) | 124.00  (109.00, 138.00) |  | 120.00  (107.00, 134.00) | 124.50  (109.75, 138.00) |
| *Z* | -1.024 | |  | -1.808 | |  | -1.808 | |  | -2.073 | |  | -1.989 | |
| *P* | 0.306 | |  | 0.071 | |  | 0.071 | |  | 0.038 | |  | 0.047 | |
| DBP (mmHg), median (IQR) | 75.50  (68.25, 83.75) | 77.00  (68.00, 85.00) |  | 75.00  (69.00, 82.00) | 77.00  (67.00, 85.00) |  | 75.00  (69.00, 82.00) | 77.00  (67.00, 85.00) |  | 75.00  (68.00, 83.00) | 77.00  (68.00, 85.00) |  | 76.00  (68.00, 85.00) | 77.00  (68.00, 83.25) |
| *Z* | -0.604 | |  | -0.753 | |  | -0.753 | |  | -0.959 | |  | -0.617 | |
| *P* | 0.546 | |  | 0.451 | |  | 0.451 | |  | 0.338 | |  | 0.537 | |
| **Gastric function indicators (n=535)** | | | | | | | | | | | | | | |
| PG-I (ng/mL), median (IQR) | 155.60  (91.31, 200.00) | 144.70  (84.83, 200.00) |  | 154.60  (98.17, 200.00) | 146.40  (84.82, 200.00) |  | 154.60  (98.17, 200.00) | 146.40  (84.82, 200.00) |  | 156.20  (98.36, 200.00) | 145.10  (84.53, 200.00) |  | 154.90  (91.41, 200.00) | 138.40  (80.56, 200.00) |
| *Z* | -1.009 | |  | -0.967 | |  | -0.967 | |  | -1.277 | |  | -1.295 | |
| *P* | 0.313 | |  | 0.334 | |  | 0.334 | |  | 0.202 | |  | 0.195 | |
| PG-II (ng/mL), median (IQR) | 21.77  (13.42, 32.84) | 14.01  (8.25, 25.99) |  | 22.90  (13.81, 33.33) | 14.95  (8.50, 26.80) |  | 22.90  (13.81, 33.33) | 14.95  (8.50, 26.80) |  | 23.05  (13.97, 34.51) | 14.30  (8.38, 26.20) |  | 20.80  (12.18, 32.57) | 13.40  (8.27, 24.33) |
| *Z* | -5.481 | |  | -4.897 | |  | -4.897 | |  | -5.852 | |  | -4.976 | |
| *P* | 0.000 | |  | 0.000 | |  | 0.000 | |  | 0.000 | |  | 0.000 | |
| PG-I/PG-II, median (IQR) | 6.42  (4.03, 9.15) | 8.51  (5.92, 12.62) |  | 6.26  (3.97, 8.81) | 8.22  (5.43, 12.15) |  | 6.26  (3.97, 8.81) | 8.22  (5.43, 12.15) |  | 6.09  (3.96, 8.59) | 8.55  (5.61, 12.40) |  | 6.81  (4.37, 9.98) | 8.74  (6.05, 12.64) |
| *Z* | -5.483 | |  | -4.782 | |  | -4.782 | |  | -5.884 | |  | -4.902 | |
| *P* | 0.000 | |  | 0.000 | |  | 0.000 | |  | 0.000 | |  | 0.000 | |
| **Missing, No. (%)** | 14 (5.93) | 16 (4.86) |  | 9 (5.49) | 21 (5.24) |  | 9 (5.49) | 21 (5.24) |  | 11 (5.88) | 19 (5.03) |  | 16 (5.21) | 14 (5.43) |
| G-17 (pg/mL) (n=554)  median (IQR) | 58.61  (24.08, 190.25) | 56.30  (18.01, 184.73) |  | 62.59  (24.76, 194.15) | 55.14  (17.95, 180.35) |  | 62.59  (24.76, 194.15) | 55.14  (17.95, 180.35) |  | 60.46  (25.68, 190.25) | 55.07  (17.80, 184.73) |  | 57.19  (23.93, 189.75) | 56.57  (16.28, 186.78) |
| *Z* | -0.834 | |  | -1.046 | |  | -1.046 | |  | -1.021 | |  | -1.171 | |
| *P* | 0.404 | |  | 0.296 | |  | 0.296 | |  | 0.307 | |  | 0.242 | |
| **Missing, No. (%)** | 4 (1.69) | 7 (2.13) |  | 3 (1.83) | 8 (2.00) |  | 3 (1.83) | 8 (2.00) |  | 3 (1.60) | 8 (2.12) |  | 5 (1.63) | 6 (2.33) |
| OB (n=414) positive**No. (%)** | 108 (62.07) | 116 (48.33) |  | 73 (59.35) | 151 (51.89) |  | 73 (59.35) | 151 (51.89) |  | 80 (59.70) | 144 (51.43) |  | 133 (61.01) | 91 (46.43) |
| *χ^2^* | 7.664 | |  | 1.937 | |  | 1.937 | |  | 2.498 | |  | 8.836 | |
| *P* | 0.006 | |  | 0.164 | |  | 0.164 | |  | 0.114 | |  | 0.003 | |
| **Missing, No. (%)** | 62 (26.27) | 89 (27.05) |  | 41 (25.00) | 110 (27.43) |  | 41 (25.00) | 110 (27.43) |  | 53 (28.34) | 98 (25.93) |  | 89 (28.99) | 62 (24.03) |
| CST4 (n=212) (U/mL) | 51.25  (32.04, 91.74) | 42.07  (23.30, 71.91) |  | 47.87  (28.90, 95.44) | 45.32  (23.84, 75.85) |  | 47.87  (28.90, 95.44) | 45.32  (23.84, 75.85) |  | 47.87  (30.73, 97.87) | 45.32  (23.74, 73.22) |  | 50.66  (31.54, 87.03) | 42.79  (22.51, 71.91) |
| *Z* | -2.385 | |  | -1.318 | |  | -1.318 | |  | -1.622 | |  | -2.203 | |
| *P* | 0.017 | |  | 0.188 | |  | 0.188 | |  | 0.105 | |  | 0.028 | |
| **Missing, No. (%)** | 136 (57.63) | 217 (65.96) |  | 95 (57.93) | 258 (64.34) |  | 95 (57.93) | 258 (64.34) |  | 106 (56.68) | 247 (65.34) |  | 191 (62.21) | 162 (62.79) |
| **Abdominal tumor markers (n=521)** | | | | | | | | | | | | | | |
| AFP (ng/mL), median (IQR) | 2.11  (1.57, 3.00) | 2.28  (1.60, 3.14) |  | 2.16  (1.63, 3.19) | 2.20  (1.57, 3.02) |  | 2.16  (1.63, 3.19) | 2.20  (1.57, 3.02) |  | 2.16  (1.60, 3.14) | 2.20  (1.58, 3.01) |  | 2.07  (1.57, 3.00) | 2.31  (1.66, 3.23) |
| *Z* | -1.032 | |  | -0.506 | |  | -0.506 | |  | -0.490 | |  | -1.515 | |
| *P* | 0.302 | |  | 0.613 | |  | 0.613 | |  | 0.624 | |  | 0.130 | |
| CEA (ng/mL), median (IQR) | 2.01  (1.44, 3.41) | 2.05  (1.35, 3.26) |  | 1.90  (1.36, 3.42) | 2.06  (1.40, 3.26) |  | 1.90  (1.36, 3.42) | 2.06  (1.40, 3.26) |  | 2.06  (1.42, 3.71) | 2.00  (1.37, 3.15) |  | 2.00  (1.36, 3.36) | 2.16  (1.40, 3.28) |
| *Z* | -0.355 | |  | -0.476 | |  | -0.476 | |  | -1.205 | |  | -0.463 | |
| *P* | 0.722 | |  | 0.634 | |  | 0.634 | |  | 0.228 | |  | 0.643 | |
| CA19-9 (U/mL), median (IQR) | 5.58  (2.92, 11.11) | 5.99  (3.13, 12.96) |  | 5.58  (2.82, 10.94) | 5.96  (3.09, 12.96) |  | 5.58  (2.82, 10.94) | 5.96  (3.09, 12.96) |  | 5.33  (2.92, 11.13) | 6.07  (3.11, 12.97) |  | 5.66  (2.91, 11.87) | 5.99  (3.20, 12.97) |
| *Z* | -0.724 | |  | -0.635 | |  | -0.635 | |  | -0.867 | |  | -0.744 | |
| *P* | 0.469 | |  | 0.525 | |  | 0.525 | |  | 0.386 | |  | 0.457 | |
| CA125 (U/mL), median (IQR) | 10.80  (7.80, 16.60) | 13.50  (8.80, 21.65) |  | 10.80  (8.10, 15.90) | 12.85  (8.40, 21.10) |  | 10.80  (8.10, 15.90) | 12.85  (8.40, 21.10) |  | 10.80  (8.40, 15.90) | 13.25  (8.38, 21.10) |  | 11.30  (8.33, 17.98) | 13.40  (8.40, 22.30) |
| *Z* | -3.401 | |  | -2.526 | |  | -2.526 | |  | -2.604 | |  | -2.676 | |
| *P* | 0.001 | |  | 0.012 | |  | 0.012 | |  | 0.009 | |  | 0.007 | |
| CA15-3 (U/mL), median (IQR) | 8.00  (5.86, 11.73) | 8.30  (6.15, 11.95) |  | 7.80  (5.80, 11.40) | 8.40  (6.10, 12.10) |  | 7.80  (5.80, 11.40) | 8.40  (6.10, 12.10) |  | 7.70  (5.60, 11.50) | 8.45  (6.30, 11.93) |  | 7.95  (5.83, 11.48) | 8.50  (6.30, 12.10) |
| *Z* | -1.102 | |  | -1.389 | |  | -1.389 | |  | -1.825 | |  | -1.643 | |
| *P* | 0.270 | |  | 0.165 | |  | 0.165 | |  | 0.068 | |  | 0.100 | |
| CA72-4 (U/mL), median (IQR) | 1.15  (0.49, 2.28) | 1.12  (0.32, 2.09) |  | 1.14  (0.50, 2.09) | 1.13  (0.35, 2.24) |  | 1.14  (0.50, 2.09) | 1.13  (0.35, 2.24) |  | 1.15  (0.51, 2.24) | 1.12  (0.34, 2.16) |  | 1.13  (0.48, 2.25) | 1.15  (0.30, 2.07) |
| *Z* | -1.022 | |  | -0.470 | |  | -0.470 | |  | -1.173 | |  | -0.777 | |
| *P* | 0.307 | |  | 0.639 | |  | 0.639 | |  | 0.241 | |  | 0.437 | |
| **Missing, No. (%)** | 20 (8.47) | 24 (7.29) |  | 13 (7.93) | 31 (7.73) |  | 13 (7.93) | 31 (7.73) |  | 16 (8.56) | 28 (7.41) |  | 23 (7.49) | 21 (8.14) |
| **Blood routine tests (n=564)** | | | | | | | | | | | | | | |
| PLT ×10^9/L, median (IQR) | 169.00  (129.00, 230.00) | 188.00  (142.50, 241.50) |  | 174.00  (131.00, 233.75) | 181.00  (137.50, 236.50) |  | 174.00  (131.00, 233.75) | 181.00  (137.50, 236.50) |  | 172.00  (136.00, 234.00) | 182.00  (138.00, 237.00) |  | 174.00  (136.00, 235.50) | 184.50  (140.50, 236.25) |
| *Z* | -2.369 | |  | -0.790 | |  | -0.790 | |  | -0.753 | |  | -0.681 | |
| *P* | 0.018 | |  | 0.430 | |  | 0.430 | |  | 0.451 | |  | 0.496 | |
| NEUT ×10^9/L, median (IQR) | 4.33  (3.06, 6.08) | 4.03  (2.84, 5.70) |  | 4.17  (2.94, 5.88) | 4.17  (2.98, 5.84) |  | 4.17  (2.94, 5.88) | 4.17  (2.98, 5.84) |  | 4.23  (3.06, 5.94) | 4.14  (2.87, 5.76) |  | 4.32  (3.09, 6.22) | 3.93  (2.76, 5.55) |
| *Z* | -1.399 | |  | -0.437 | |  | -0.437 | |  | -0.849 | |  | -2.397 | |
| *P* | 0.162 | |  | 0.662 | |  | 0.662 | |  | 0.396 | |  | 0.017 | |
| LYC, median (IQR) | 1.31  (0.96, 1.75) | 1.32  (0.98, 1.77) |  | 1.31  (0.97, 1.75) | 1.32  (0.97, 1.74) |  | 1.31  (0.97, 1.75) | 1.32  (0.97, 1.74) |  | 1.35  (1.03, 1.75) | 1.30  (0.96, 1.75) |  | 1.32  (0.95, 1.75) | 1.31  (1.00, 1.74) |
| *Z* | -0.120 | |  | -0.624 | |  | -0.624 | |  | -1.035 | |  | -0.215 | |
| *P* | 0.905 | |  | 0.532 | |  | 0.532 | |  | 0.300 | |  | 0.830 | |
| PLR, median (IQR)) | 128.57  (91.58, 192.77) | 138.83  (100.00, 204.45) |  | 128.99  (84.16, 191.11) | 136.33  (99.67, 204.67) |  | 128.99  (84.16, 191.11) | 136.33  (99.67, 204.67) |  | 128.44  (91.94, 190.44) | 138.75  (99.37, 207.32) |  | 133.63  (95.93, 202.88) | 134.83  (99.40, 194.22) |
| *Z* | -1.350 | |  | -1.486 | |  | -1.486 | |  | -1.529 | |  | -0.133 | |
| *P* | 0.177 | |  | 0.137 | |  | 0.137 | |  | 0.126 | |  | 0.894 | |
| NLR,median (IQR) | 3.01  (2.20, 4.80) | 3.15  (2.00, 4.99) |  | 2.85  (2.11, 4.59) | 3.16  (2.11, 5.01) |  | 2.85  (2.11, 4.59) | 3.16  (2.11, 5.01) |  | 2.97  (2.20, 4.70) | 3.16  (2.02, 5.03) |  | 3.09  (2.24, 5.05) | 3.10  (1.90, 4.82) |
| *Z* | -0.663 | |  | -1.063 | |  | -1.063 | |  | -0.100 | |  | -1.472 | |
| *P* | 0.507 | |  | 0.288 | |  | 0.288 | |  | 0.921 | |  | 0.141 | |
| **Missing, No. (%)** | 1 (0.42) | 0 (0.00) |  | 0 (0.00) | 1 (0.25) |  | 0 (0.00) | 1 (0.25) |  | 0 (0.00) | 1 (0.26) |  | 1 (0.33) | 0 (0.00) |
| FBG (n=546) (mmol/L)  median (IQR) | 5.40  (4.80, 6.70) | 5.30  (4.80, 6.30) |  | 5.30  (4.80, 6.45) | 5.30  (4.80, 6.50) |  | 5.30  (4.80, 6.45) | 5.30  (4.80, 6.50) |  | 5.40  (4.80, 6.60) | 5.30  (4.80 6.40) |  | 5.40  (4.80, 6.70) | 5.30  (4.70, 6.30) |
| *Z* | -1.031 | |  | -0.025 | |  | -0.025 | |  | -0.881 | |  | -1.771 | |
| *P* | 0.303 | |  | 0.980 | |  | 0.980 | |  | 0.379 | |  | 0.076 | |
| **Missing, No. (%)** | 5 (2.12) | 14 (4.26) |  | 3 (1.83) | 16 (3.99) |  | 3 (1.83) | 16 (3.99) |  | 4 (2.14) | 15 (3.97) |  | 8 (2.61) | 11 (4.26) |
| **Serum lipid parameters (n=284)** | | | | | | | | | | | | | | |
| GSP (μmol/L), median (IQR) | 170.00  (146.00, 210.00) | 179.00  (150.50, 202.00) |  | 169.00  (145.50, 208.50) | 179.00  (149.00, 203.00) |  | 169.00  (145.50, 208.50) | 179.00  (149.00, 203.00) |  | 166.00  (144.00, 198.00) | 181.00  (154.50, 204.00) |  | 170.00  (144.00, 198.00) | 186.00  (156.00, 206.50) |
| *Z* | -0.520 | |  | -0.482 | |  | -0.482 | |  | -1.602 | |  | -2.168 | |
| *P* | 0.603 | |  | 0.629 | |  | 0.629 | |  | 0.109 | |  | 0.030 | |
| TC(mmol/L), median (IQR) | 3.65  (3.10, 4.51) | 3.86  (3.15, 4.63) |  | 3.54  (3.02, 4.51) | 3.81  (3.16, 4.63) |  | 3.54  (3.02, 4.51) | 3.81  (3.16, 4.63) |  | 3.60  (3.08, 4.51) | 3.81  (3.14, 4.63) |  | 3.64  (3.04, 4.63) | 3.84  (3.19, 4.62) |
| *Z* | -1.261 | |  | -1.367 | |  | -1.367 | |  | -1.195 | |  | -1.145 | |
| *P* | 0.207 | |  | 0.171 | |  | 0.171 | |  | 0.232 | |  | 0.252 | |
| TG(mmol/L), median (IQR) | 1.36  (0.94, 2.12) | 1.35  (0.94, 1.78) |  | 1.34  (0.91, 2.12) | 1.37  (0.94, 1.84) |  | 1.34  (0.91, 2.12) | 1.37  (0.94, 1.84) |  | 1.34  (0.94, 2.12) | 1.40  (0.93, 1.85) |  | 1.35  (0.94, 1.89) | 1.38  (0.94, 1.86) |
| *Z* | -0.929 | |  | -0.446 | |  | -0.446 | |  | -0.707 | |  | -0.278 | |
| *P* | 0.353 | |  | 0.656 | |  | 0.656 | |  | 0.480 | |  | 0.781 | |
| HDL-C (mmol/L), median (IQR) | 0.83  (0.59, 1.07) | 0.85  (0.65, 1.15) |  | 0.82  (0.60, 1.07) | 0.86  (0.64, 1.14) |  | 0.82  (0.60, 1.07) | 0.86  (0.64, 1.14) |  | 0.82  (0.57, 1.06) | 0.86  (0.65, 1.16) |  | 0.82  (0.60, 1.02) | 0.88  (0.67, 1.19) |
| *Z* | -0.905 | |  | -0.895 | |  | -0.895 | |  | -1.501 | |  | -1.922 | |
| *P* | 0.365 | |  | 0.371 | |  | 0.371 | |  | 0.133 | |  | 0.055 | |
| LDL-C (mmol/L), median (IQR) | 1.95  (1.39, 2.61) | 2.18  (1.62, 2.82) |  | 1.92  (1.34, 2.60) | 2.16  (1.62, 2.76) |  | 1.92  (1.34, 2.60) | 2.16  (1.62, 2.76) |  | 1.95  (1.39, 2.64) | 2.16  (1.57, 2.77) |  | 2.01  (1.43, 2.72) | 2.16  (1.67, 2.72) |
| *Z* | -1.895 | |  | -2.044 | |  | -2.044 | |  | -1.328 | |  | -1.104 | |
| *P* | 0.058 | |  | 0.041 | |  | 0.041 | |  | 0.184 | |  | 0.270 | |
| THCY (μmol/L), median (IQR) | 12.60  (10.40, 15.80) | 13.50  (10.55, 16.85) |  | 12.90  (10.30, 15.40) | 13.30  (10.40, 16.90) |  | 12.90  (10.30, 15.40) | 13.30  (10.40, 16.90) |  | 12.90  (10.50, 15.80) | 13.30  (10.35, 16.80) |  | 12.60  (10.40, 15.80) | 13.90  (10.70, 16.90) |
| *Z* | -1.088 | |  | -0.951 | |  | -0.951 | |  | -0.662 | |  | -1.638 | |
| *P* | 0.277 | |  | 0.342 | |  | 0.342 | |  | 0.508 | |  | 0.101 | |
| **Missing, No. (%)** | 121 (51.27) | 160 (48.63) |  | 83 (50.61) | 198 (49.38) |  | 83 (50.61) | 198 (49.38) |  | 100 (53.48) | 181 (47.88) |  | 156 (50.81) | 125 (48.45) |
| **Liver and kidney function indicators (n=558)** | | | | | | | | | | | | | | |
| Cr (µmol/L), median (IQR) | 71.00  (57.00, 84.00) | 67.00  (55.00, 84.00) |  | 70.00  (56.00, 83.00) | 68.00  (56.00, 84.75) |  | 70.00  (56.00, 83.00) | 68.00  (56.00, 84.75) |  | 71.00  (56.50, 84.00) | 68.00  (56.00, 84.00) |  | 71.00  (58.00, 84.00) | 66.00  (55.00, 84.00) |
| *Z* | -1.015 | |  | -0.022 | |  | -0.022 | |  | -0.446 | |  | -1.573 | |
| *P* | 0.310 | |  | 0.982 | |  | 0.982 | |  | 0.656 | |  | 0.116 | |
| ALT (U/L), median (IQR) | 19.00  (13.00, 30.50) | 18.00  (13.00, 26.00) |  | 19.00  (13.00, 30.00) | 18.00  (13.00, 27.00) |  | 19.00  (13.00, 30.00) | 18.00  (13.00, 27.00) |  | 20.00  (13.00, 29.50) | 18.00  (13.00, 26.00) |  | 18.00  (13.00, 28.00) | 18.00  (13.00, 26.00) |
| *Z* | -1.088 | |  | -0.815 | |  | -0.815 | |  | -1.289 | |  | -0.258 | |
| *P* | 0.277 | |  | 0.415 | |  | 0.415 | |  | 0.197 | |  | 0.796 | |
| AST (U/L), median (IQR) | 22.00  (18.00, 29.00) | 22.00  (18.00, 28.00) |  | 22.00  (17.75, 29.00) | 22.00  (18.00, 28.00) |  | 22.00  (17.75, 29.00) | 22.00  (18.00, 28.00) |  | 22.00  (18.00, 29.00) | 22.00  (18.00, 28.00) |  | 22.00  (18.00, 28.00) | 23.00  (18.00, 29.00) |
| *Z* | -0.652 | |  | -0.160 | |  | -0.160 | |  | -0.456 | |  | -0.469 | |
| *P* | 0.514 | |  | 0.873 | |  | 0.873 | |  | 0.648 | |  | 0.639 | |
| GGT (U/L), median (IQR) | 16.00  (11.00, 25.00) | 17.00  (11.50, 27.00) |  | 16.00  (11.00, 25.75) | 17.00  (12.00, 27.00) |  | 16.00  (11.00, 25.75) | 17.00  (12.00, 27.00) |  | 16.00  (11.00, 28.00) | 16.00  (11.00, 27.00) |  | 16.00  (11.00, 27.00) | 17.00  (11.00, 27.00) |
| *Z* | -1.039 | |  | -1.160 | |  | -1.160 | |  | -0.130 | |  | -0.704 | |
| *P* | 0.299 | |  | 0.246 | |  | 0.246 | |  | 0.897 | |  | 0.481 | |
| TBIL (µmol/L), median (IQR) | 13.00  (9.00, 18.50) | 13.20  (9.20, 18.00) |  | 13.00  (9.80, 17.33) | 14.00  (9.00, 18.80) |  | 13.00  (9.80, 17.33) | 14.00  (9.00, 18.80) |  | 13.00  (9.00, 18.00) | 14.00  (9.00, 18.00) |  | 13.00  (9.00, 18.20) | 13.50  (10.00, 18.00) |
| *Z* | -0.114 | |  | -0.798 | |  | -0.798 | |  | -0.733 | |  | -0.363 | |
| *P* | 0.909 | |  | 0.425 | |  | 0.425 | |  | 0.464 | |  | 0.717 | |
| **Missing, No. (%)** | 3 (1.27) | 4 (1.22) |  | 2 (1.22) | 5 (1.25) |  | 2 (1.22) | 5 (1.25) |  | 2 (1.07) | 5 (1.32) |  | 4 (1.30) | 3 (1.16) |
| **Coagulation function parameters (n=520)** | | | | | | | | | | | | | | |
| PT (s), median (IQR) | 10.80  (10.20, 11.40) | 10.80  (10.30, 11.65) |  | 10.80  (10.10, 11.60) | 10.80  (10.28, 11.50) |  | 10.80  (10.10, 11.60) | 10.80  (10.28, 11.50) |  | 10.80  (10.20, 11.60) | 10.80  (10.20, 11.50) |  | 10.80  (10.20, 11.50) | 10.80  (10.30, 11.50) |
| *Z* | -1.350 | |  | -0.676 | |  | -0.676 | |  | -0.045 | |  | -0.267 | |
| *P* | 0.177 | |  | 0.499 | |  | 0.499 | |  | 0.965 | |  | 0.790 | |
| PT%, median (IQR) | 94.70  (88.50, 103.20) | 92.60  (84.40, 100.80) |  | 94.30  (88.33, 103.80) | 93.40  (85.10, 100.80) |  | 94.30  (88.33, 103.80) | 93.40  (85.10, 100.80) |  | 93.90  (87.30, 103.20) | 93.40  (85.10, 100.80) |  | 93.40  (86.70, 102.95) | 93.40  (85.10, 100.80) |
| *Z* | -2.255 | |  | -1.591 | |  | -1.591 | |  | -1.250 | |  | -1.293 | |
| *P* | 0.024 | |  | 0.112 | |  | 0.112 | |  | 0.211 | |  | 0.196 | |
| PT-INR, median (IQR) | 0.92  (0.87, 0.97) | 0.93  (0.88, 1.00) |  | 0.92  (0.87, 0.97) | 0.93  (0.88, 1.00) |  | 0.92  (0.87, 0.97) | 0.93  (0.88, 1.00) |  | 0.93  (0.87, 0.97) | 0.92  (0.88, 1.00) |  | 0.93  (0.87, 0.98) | 0.92  (0.88, 1.00) |
| *Z* | -2.198 | |  | -1.311 | |  | -1.311 | |  | -0.756 | |  | -0.899 | |
| *P* | 0.028 | |  | 0.190 | |  | 0.190 | |  | 0.450 | |  | 0.369 | |
| APTT (s), median (IQR) | 26.20  (23.90, 28.30) | 26.80  (24.60, 29.15) |  | 26.50  (23.58, 28.53) | 26.50  (24.50, 29.00) |  | 26.50  (23.58, 28.53) | 26.50  (24.50, 29.00) |  | 26.30  (23.60, 28.50) | 26.50  (24.60, 29.00) |  | 26.25  (23.90, 28.50) | 26.65  (24.80, 29.10) |
| *Z* | -2.123 | |  | -1.300 | |  | -1.300 | |  | -1.543 | |  | -1.904 | |
| *P* | 0.034 | |  | 0.194 | |  | 0.194 | |  | 0.123 | |  | 0.057 | |
| FIB (g/L), median (IQR) | 2.89  (2.37, 3.68) | 3.01  (2.37, 3.74) |  | 3.04  (2.45, 3.69) | 2.90  (2.32, 3.70) |  | 3.04  (2.45, 3.69) | 2.90  (2.32, 3.70) |  | 2.99  (2.36, 3.76) | 2.94  (2.39, 3.68) |  | 2.93  (2.34, 3.70) | 2.99  (2.42, 3.70) |
| *Z* | -0.754 | |  | -1.049 | |  | -1.049 | |  | -0.011 | |  | -0.489 | |
| *P* | 0.451 | |  | 0.294 | |  | 0.294 | |  | 0.991 | |  | 0.625 | |
| TT (s), median (IQR) | 17.80  (16.90, 18.80) | 17.90  (17.00, 18.70) |  | 17.60  (16.80, 18.63) | 17.90  (17.10, 18.70) |  | 17.60  (16.80, 18.63) | 17.90  (17.10, 18.70) |  | 17.70  (16.80, 18.70) | 17.90  (17.10, 18.70) |  | 17.80  (16.90, 18.78) | 17.90  (17.00, 18.70) |
| *Z* | -0.785 | |  | -1.870 | |  | -1.870 | |  | -1.971 | |  | -0.664 | |
| *P* | 0.432 | |  | 0.062 | |  | 0.062 | |  | 0.049 | |  | 0.507 | |
| D-D (μg/mL), median (IQR) | 0.31  (0.17, 0.73) | 0.41  (0.20, 1.04) |  | 0.31  (0.17, 0.66) | 0.41  (0.19, 1.03) |  | 0.31  (0.17, 0.66) | 0.41  (0.19, 1.03) |  | 0.34  (0.18, 0.73) | 0.41  (0.19, 1.02) |  | 0.35  (0.18, 0.81) | 0.41  (0.21, 1.03) |
| *Z* | -2.468 | |  | -2.674 | |  | -2.674 | |  | -1.804 | |  | -2.193 | |
| *P* | 0.014 | |  | 0.007 | |  | 0.007 | |  | 0.071 | |  | 0.028 | |
| **Missing, No. (%)** | 17 (7.20) | 28 (8.51) |  | 10 (6.10) | 35 (8.73) |  | 10 (6.10) | 35 (8.73) |  | 12 (6.42) | 33 (8.73) |  | 27 (8.79) | 18 (6.98) |


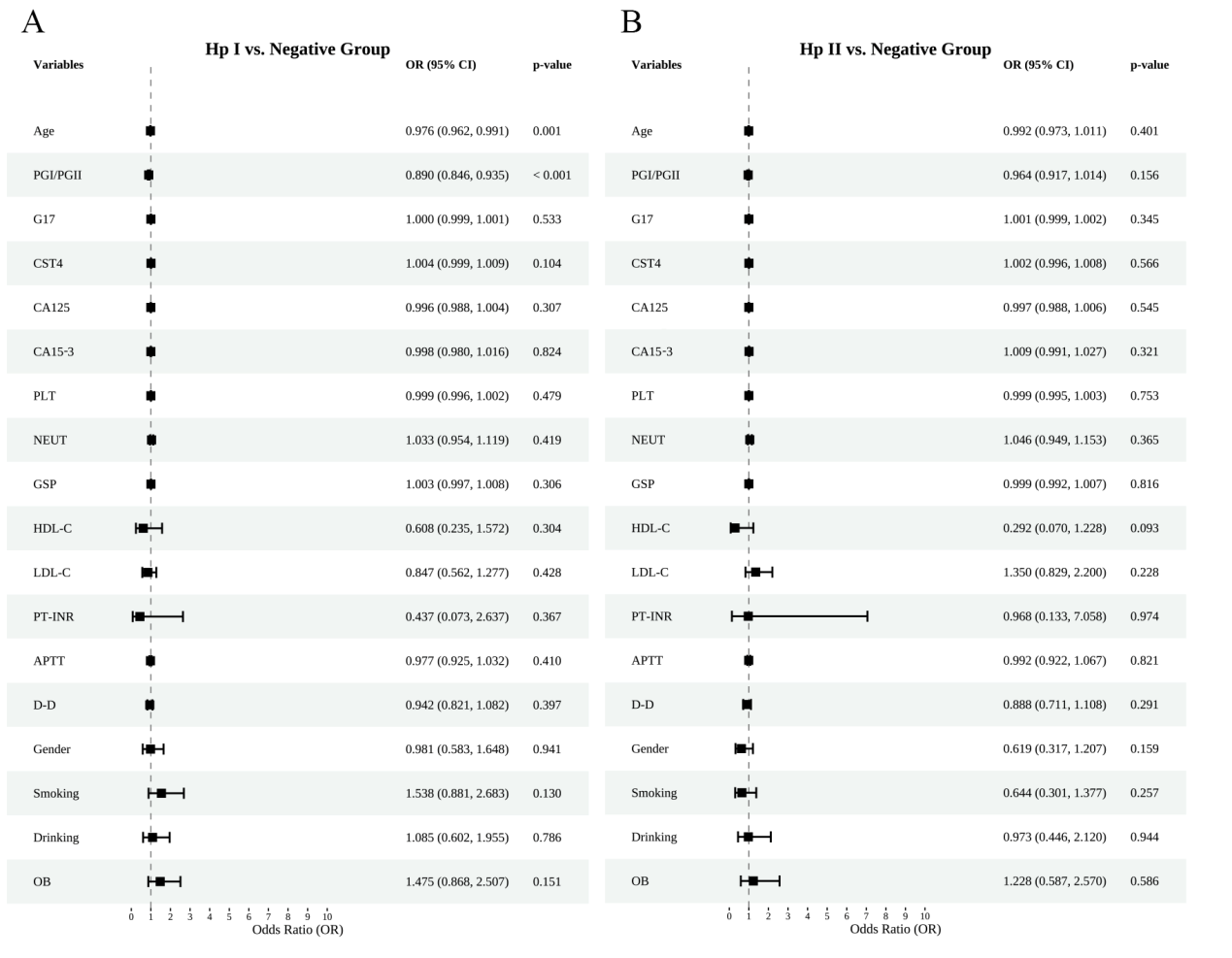


**Supplementary Figure 1. Multivariate logistic analyses of risk factors.** (A) Risk factors associated with the Hp I infection group (n = 240). (B) Risk factors associated with the Hp II group (n = 81). Note: A P-value less than 0.05 indicates a statistically significant difference.


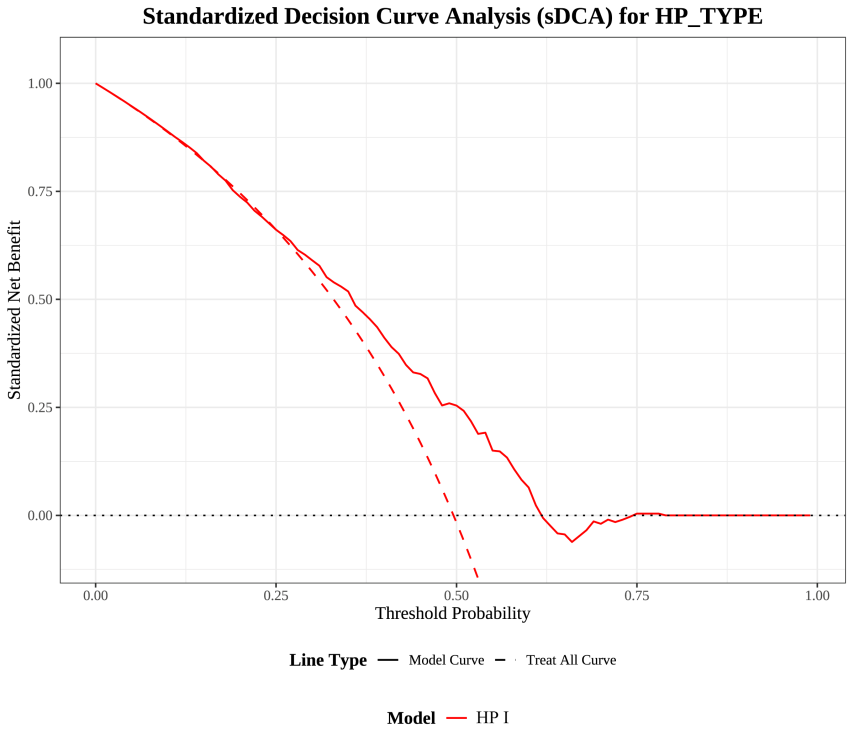


**Supplementary Figure 2. sDCA evaluating the clinical utility of the nomogram.**sDCA for the nomogram predicting Hp I infection.


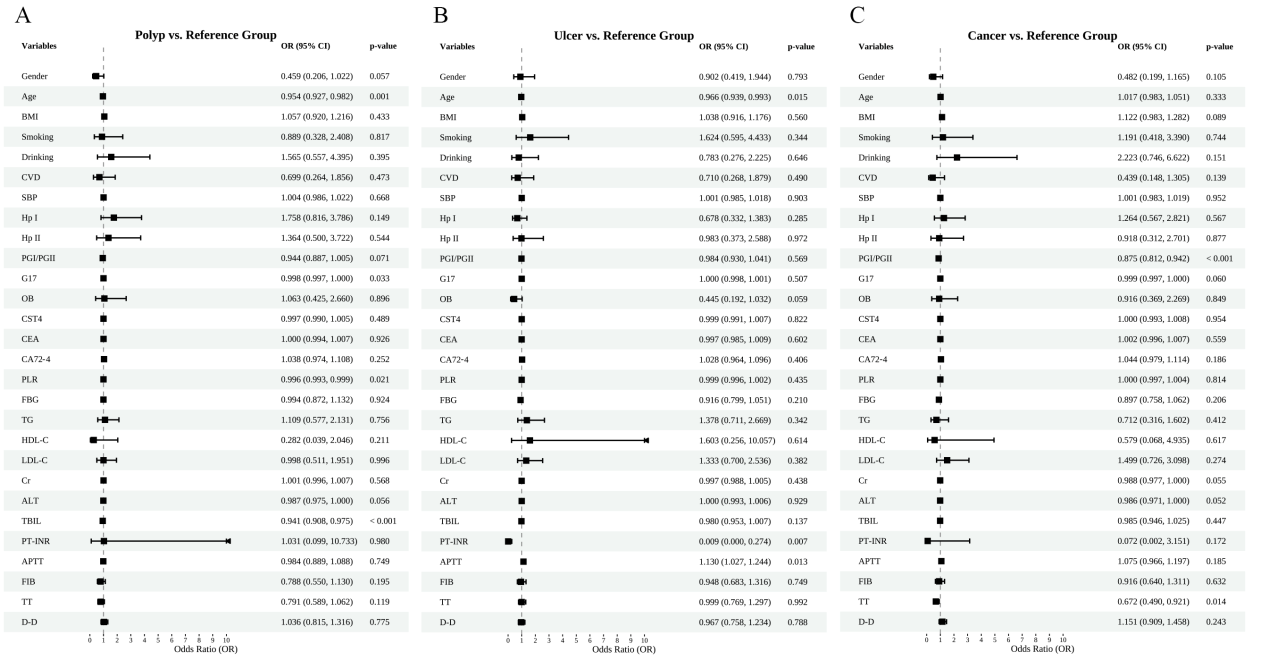


**Supplementary Figure 3. Forest plots of multivariable logistic regression analyses.** The polyp (A), ulcer (B), and cancer (C) groups versus a reference group. OR with 95% CI are shown for each variable. Significant associations (*P* < 0.05) are highlighted.


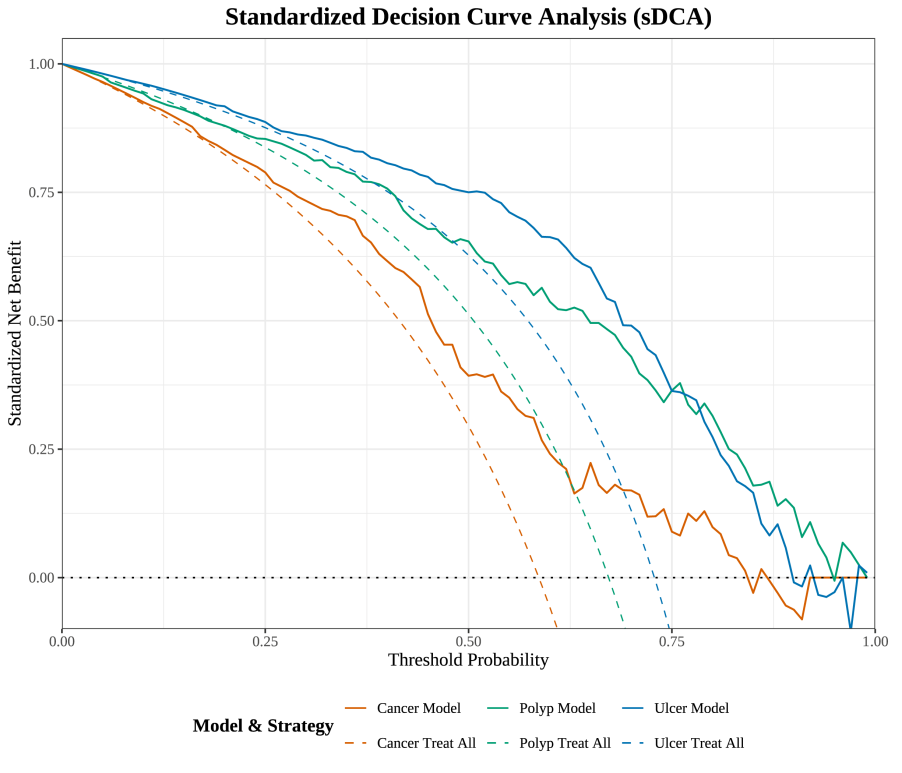


**Supplementary Figure 4. Evaluating Clinical Utility of Gastric Lesion Prediction Models via sDCA.** Three risk‑stratified models provide higher net clinical benefit than the "treat-all" strategy across most decision thresholds, validating their use in personalized clinical guidance.
